# Porous Polymer Materials in Triboelectric Nanogenerators: A Review

**DOI:** 10.3390/polym15224383

**Published:** 2023-11-11

**Authors:** Yajun Mi, Zequan Zhao, Han Wu, Yin Lu, Ning Wang

**Affiliations:** 1 Center for Green Innovation, School of Mathematics and Physics, University of Science and Technology Beijing, Beijing 100083, China; d202110423@xs.ustb.edu.cn (Y.M.); m202110789@xs.ustb.edu.cn (Z.Z.); b20200369@xs.ustb.edu.cn (Y.L.); 2 National Electronic Computer Quality Inspection and Testing Center, Beijing 100083, China; wuhan@cetec.org.cn

**Keywords:** porous polymers, triboelectric nanogenerator, energy harvester, self-powered sensor, multifunctionality

## Abstract

Since the invention of the triboelectric nanogenerator (TENG), porous polymer materials (PPMs), with different geometries and topologies, have been utilized to enhance the output performance and expand the functionality of TENGs. In this review, the basic characteristics and preparation methods of various PPMs are introduced, along with their applications in TENGs on the basis of their roles as electrodes, triboelectric surfaces, and structural materials. According to the pore size and dimensionality, various types of TENGs that are built with hydrogels, aerogels, foams, and fibrous media are classified and their advantages and disadvantages are analyzed. To deepen the understanding of the future development trend, their intelligent and multifunctional applications in human–machine interfaces, smart wearable devices, and self-powering sensors are introduced. Finally, the future directions and challenges of PPMs in TENGs are explored to provide possible guidance on PPMs in various TENG-based intelligent devices and systems.

## 1. Introduction

The integration of artificial intelligence (AI) and the Internet of Things (IoTs) is spawning rising demand for small, flexible, and portable systems that are built with renewable power sources for sustainable operation [1,2,3]. Emerging as the time requires, the TENG was invented for harvesting ambient mechanical energy that would otherwise be wasted and converts it into usable alternating current for continuously powering portable systems [4,5]. Compared to other renewable energy harvesting technologies such as solar and thermoelectric systems, TENGs have advantages in structure adaptivity, convenience of materials selection, as well as reversibility in sensing both the chemical and physical variation at the triboelectric interface [6]. By collecting energy from environmental vibration sources, as it is easily accessible in the surrounding environment as well as from our own human movements, they successfully find wide applications in high-voltage power supplies, biomedical devices, speech recording, wind speed monitoring, blue energy harvesting, and other self-powered intelligent systems [7,8,9,10,11].

Up to this point, the TENG has experienced a swift and thriving phase of growth. This encompasses everything from the intricacies of structural planning, the meticulous selection and refinement of materials, to the fine-tuning of performance, adept power management, and the dynamic exploration of applications [12,13]. Various successful approaches have been implemented to enhance the output attributes of the TENG, ensuring it meets the diverse demands for energy provision and tactile sensing across a range of applications [9,14]. For example, selecting appropriate materials, optimizing structural design, improving preparation processes, and introducing external fields. A common method to enhance TENG output performance is to introduce micro- and nano-scale morphology on the surface of triboelectric materials to increase the electrification area. However, this process often involves high-cost technologies such as anodizing, laser-assisted processing, etching, and micro-printing, which seriously hind its practical applications [15,16,17]. Additionally, intrinsic defects of dielectrics and electrodes restrict the multifunctionality of TENGs. These issues include mechanical defects such as a lack of stretchability and flexibility, limited contact area, and insufficient breathability [14,18]. Meanwhile, some specific applications require custom triboelectric materials to meet diverse characteristics and multifunctional demands. For instance, in biomedical applications, self-powered sensors that need to be mounted on living organs might require porous dielectrics to enhance breathability. In response to these challenges and requirements, there has been a notable surge of interest in the development of next-generation high-performance TENGs based on porous designs. These designs encompass both porous material and structural configurations [19,20,21].

Porous structures exhibit notable permeability for gases and liquids [22,23,24,25,26]. In this context, materials featuring a highly porous network exhibit exceptional qualities in terms of mechanical, electrical, thermal, optical, and surface properties. Porous materials can manifest in diverse forms, including aerogels, hydrogels, fibers, and foams [27,28,29]. Hydrogels are materials formed due to the swelling of water within a gel [30,31,32]. Aerogels are a unique form of hydrogel where the internal liquid is replaced by gas [33,34,35]. Fiber media of different sizes intertwine in a non-woven arrangement, providing internal pores [36,37,38,39]. Foams are defined as highly compressed gases dispersed in solids or liquids [40,41,42]. Due to the characteristics of the void units, these porous materials exhibit a wide range of exceptional properties [43]. At the same time, PPMs also possess a large specific surface area and specific volume, making them an ideal choice for membranes requiring high roughness and various applications. These diverse characteristics expand the potential of porous materials in various application fields, including thermal resistance, electromagnetic interference shielding, filtration, and high-magnetic-permeability films. The customizability and tunability of these materials allow them to meet the unique demands of different applications. The preparation and application of porous dielectrics are actively being researched, especially in the case of hydrogels and fiber materials. Abundant evidence has shown that the number and size of pores are crucial for defining the behavior of materials. Additionally, the porous structure of PPMs not only creates a rough surface but also offers an immense internal surface area, which might be beneficial for generating additional charges [44,45].

At the same time, electrostatic induction plays a crucial role in shaping the output characteristics of the TENG, wherein the TENG is conceptualized as a parallel plate capacitor comprising a dielectric layer and electrodes [46]. It is a widely acknowledged principle that the charge in a capacitor is directly linked to the surface area and inversely related to the separation between its upper and lower plates. Hence, diminishing the thickness of the dielectric layer presents another avenue for augmenting the output efficiency of TENGs. In addition to enhancing performance, advancements have been made in augmenting features like nanomechanical and optical properties. Consequently, there has been a notable shift in research focus towards incorporating porous dielectrics and electrodes in TENG design [47,48]. Thus, it is essential to summarize the latest literature on the development of TENGs on the basis of PPMs for further studies.

**Figure 1:**
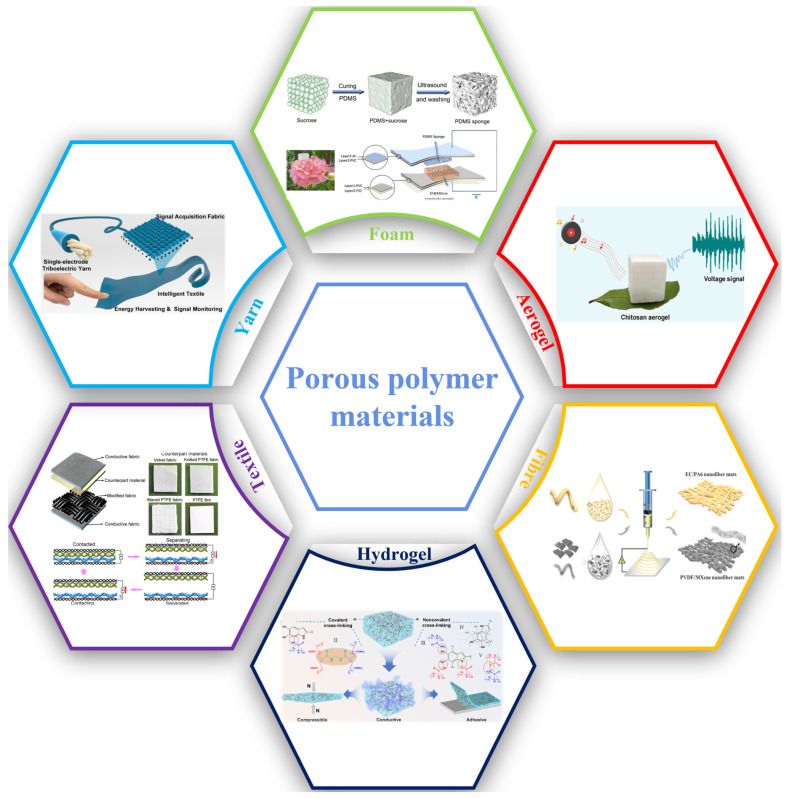
Schematic diagram of the classification of PPMs proposed in this review for PPMTENGs, including foams, Reproduced with permission [43]. Copyright 2023, American Chemical Society. aerogels, Reproduced with permission [44]. Copyright 2023, American Chemical Society. fibers, Reproduced with permission [45]. Copyright 2021, American Chemical Society. hydrogels, Reproduced with permission [46]. Copyright 2023, American Chemical Society. textiles, Reproduced with permission [47]. Copyright 2021, American Chemical Society. and yarns. Reproduced with permission [48]. Copyright 2020, American Chemical Society.

Thus, in this review, we aim to offer a comprehensive discussion on the development of porous polymer dielectrics and electrodes in PPMTENGs. By delving deep into the research progress and application domains of porous structural design to fully grasp their roles and advantages in the fabrication of PPMTENGs, the article explores the extensive prospects required for the performance enhancement of PPMTENGs, with a specific emphasis on the significance of the pore size. This review also summarizes various types of aerogels, foams, fiber membranes, and hydrogel polymer materials, as well as their composites with high-dielectric fillers, highlighting their respective performance characteristics. Additionally, discussions on strategies that try to realize the practical and reliable applications in actual operational settings are undertaken. In conclusion, the future developments and prospects of PPMs in TENG applications are concisely illustrated (Figure 1).

## 2. Basic Principle and Working Mode of TENG

**Figure 2:**
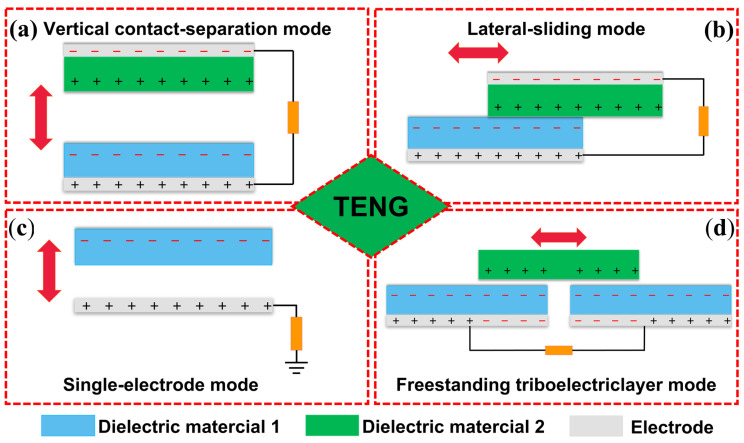
Four fundamental working modes of TENG: (**a**) Vertical contact separation mode. (**b**) Lateral sliding mode. (**c**) Single-electrode mode. (**d**) Freestanding triboelectric layer mode.

Since its invention in 2012, many TENG designs have been developed, which can be used to collect various types of mechanical energy based on triboelectric charging and electrostatic induction coupling [49]. When subjected to mechanical force, two triboelectric layers make contact, resulting in the generation of triboelectric surface charges. Upon the release of the mechanical force, the layers are separated, leading to the establishment of a potential difference. Metal electrodes on the outer surface of the triboelectric layer hold electrostatic induction charges. These induced charges flow through the electrodes to neutralize the established potential difference, giving rise to intermittent current pulses through successive cycles of contact and separation. Based on the different movement modes of the triboelectric layer and the different configurations of the electrodes, many research groups have achieved four basic operation modes of the TENG (Figure 2), including (i) vertical contact separation mode; (ii) lateral sliding mode; (iii) single-electrode mode; and (iv) independent mode.

Vertical contact separation mode: It consists of two triboelectric layers separated by space vertically (Figure 2a). When external mechanical force is applied, one layer physically contacts the other layer in the vertical direction at the interface, and the two triboelectric layers with different electric polarities come into contact with each other, resulting in opposite charges on the interface between the two triboelectric layers. Then, when the applied force is released, the two surfaces separate, resulting in a potential drop between the two electrodes, and the potential difference between the two triboelectric layers drives electrons through an external load. Through the cycle of contact separation, the potential between the two electrodes changes, causing the electrons to flow forward and backward, generating an AC-type current [50].

Lateral sliding mode: It has a structure similar to that of the contact separation mode, but it consists of two triboelectric layers that slide relative to each other, based on the triboelectric surface charges generated by the relative lateral movement between the contact surfaces (Figure 2b). Under external force, the triboelectric layers begin to slide outwards, causing changes in the contact surface area and the potential difference between the electrodes. Then, electrons flow from one electrode to the other to reduce the potential difference, resulting in the generation of current. When the triboelectric layers slide back, the contact surface area changes again, causing a change in the potential difference between the electrodes, leading to the transfer of electron backflow. Mechanical movements such as rotation of disks and cylinders and planar motion can cause sliding. The TENG can also generate energy during stretching and has been successfully applied in this mode [51].

Single-electrode mode: It is based on a single electrode covered by a triboelectric layer or only a single electrode connected to the ground with a freely movable charged object (Figure 2c). Once the freely movable object contacts the electrode, charges can be generated on the interface. In the process of movement, the potential distribution in the single-electrode mode will change with the variation of the charged surface, and electrons will flow from the ground to the electrode and back, thus generating electricity. This mode can function autonomously and without reliance on a spacer to isolate the two triboelectric layers. The uncomplicated setup, comprising a solitary dielectric layer and an electrode devoid of any intervening components, facilitates the realization of a flexible or self-repairing TENG [52].

Freestanding triboelectric layer mode: It consists of a freely movable triboelectric object and two fixed electrodes (Figure 2d). Once the freestanding triboelectric layer contacts or separates from the electrodes, the potential distribution will change, thereby promoting the flow of current. This mode has the advantages of device integration and wear resistance, as charge transfer can be achieved without physical contact. Different types of TENG have their unique advantages and application scenarios. TENGs inspired by the skin usually adopt single-electrode mode, which is widely reported due to its simplicity and easy implementation [53].

## 3. Porous Polymer Materials and Structures in TENG Design

**Figure 3:**
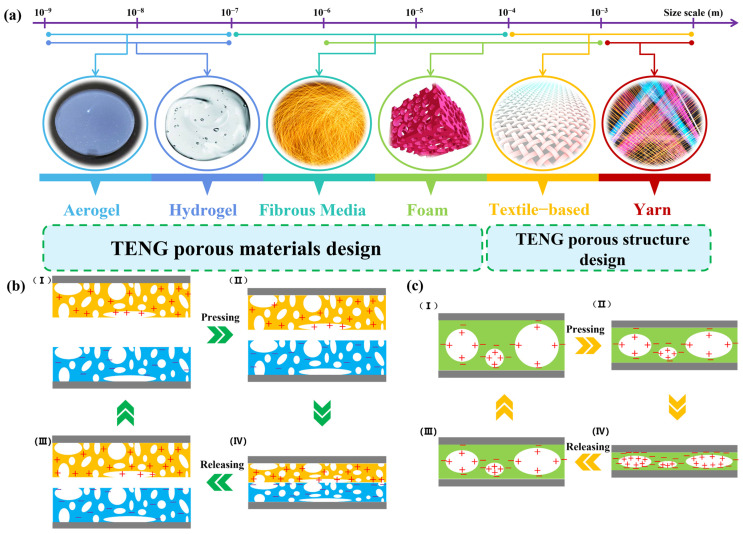
(**a**) Various porous designs of TENGs: (**left**) material designs, including aerogel, hydrogel, fibrous medium, and foam; (**right**) structural designs, including textiles and yarns; (**b**) the working cycle of TENG in the contact separation mode, and (**c**) the effect of the air-gap cell on the triboelectric performance of the gapless TENG.

A porous TENG is achieved by adopting PPMs (such as aerogels, hydrogels, foams, and fibers) or by designing porous structures (like textiles and yarns). Based on the material preparation method and structural design strategy, different ranges of pore sizes and dimensions can be obtained [54,55]. Generally, the pore sizes of porous materials can typically be divided into three main groups: micropores (pore diameter less than 2 nm), mesopores (pore diameter between 2 nm and 50 nm), and macropores (pore diameter greater than 50 nm). However, due to the broad range of porous materials and structures used for TENGs, ranging from a few nm (as in the case of gels) to macro mm (as in textiles and yarns), the porous designs can be categorized into five classes: ultramicropores (less than 1 nm), nanopores (1 nm to 1 mm), micropores (1 mm to 62.5 μm), mesopores (62.5 μm to 4 mm), and macropores (4 mm to 256 mm). The TENG materials and structures discussed in this article exhibit a range of pore sizes, spanning from 1 nm to 1 cm. Figure 3 visually depicts the diversity in pore sizes among various materials and structural configurations. PPMTENGs can be categorized based on the design of various porous materials, which include hydrogels, aerogels, fibrous media, and foams, or they can be classified based on structural design or assembly methods, such as those based on textiles and yarns [56]. Additionally, natural PPMs like wood and bamboo have also been employed in TENG technology [57,58].

To delve deeper into this field, we first discuss porous dielectrics and electrodes as material pathways for PPMTENGs, followed by an exploration of porous structural design. From a material perspective, regardless of the operational mode of the TENG, PPMs and, in some cases, other inorganic and composite materials because of their interwovenness with polymers, with outstanding physical, mechanical, thermal, and electrical properties, can be used as dielectrics and electrodes in TENG fabrication. Most porous hydrogel materials are synthesized in aqueous environments, and to maintain their hydration, they are widely used in TENG structures as electrodes. Thus, we first provide a review on polymer porous dielectrics (including aerogels, foams, and fibrous media), followed by a review on porous electrodes (based on hydrogels and to some extent based on fibrous media). Subsequently, we discuss porous structural designs, including yarn structures in monolithic TENGs, as well as textile-based structures with embroidered or knitted TENG shapes, all of which incorporate active materials and electrodes.

### 3.1. Porous Polymer Dielectric Materials

Dielectrics are a class of materials with specific electrical properties. They can conduct electric charges under the influence of an electric field, but they restrict the movement of charges over relatively long distances. Among them, the contact area (S) and the surface charge density (σ) are two key features of the dielectric, which together determine the amount of charge the material surface can accommodate [59,60]. As the dielectric participates in the TENG cycle, these charges migrate between the dielectrics. When an external force is applied, charges of opposite polarity are induced within the dielectric. In addition to the contact area and surface charge density, the dielectric constant (also known as the relative dielectric constant, denoted as ε) is another important material parameter affecting TENG performance. The dielectric constant describes the dielectric’s electric field response capability relative to a vacuum or air. The higher it is, the stronger the ability of the dielectric to store charges in an electric field [61,62]. Dielectrics with high dielectric constants can effectively store and release charges during the TENG cycle. When the two dielectrics separate during the TENG cycle, induced charges lead to the flow of free electrons, thereby generating induced voltage and current in the external circuit. This process provides the foundation for the electrical energy output of the TENG, and the magnitude of the output voltage and current depends on many factors, including, but not limited to, contact area, surface charge density, and dielectric constant [63,64]. Through a comprehensive analysis of existing literature, we can conclude that the electrical properties of dielectrics are crucial for TENG performance. Therefore, when designing and selecting dielectric materials, parameters such as contact area, surface charge density, and dielectric constant must be considered comprehensively to achieve optimized TENG system design and performance enhancement.

The unique structure of PPMs gives them a larger surface area relative to their volume, providing them with excellent contact properties [29,65]. In TENG systems, the use of porous dielectrics has become an important research direction. Researchers have explored various physical, chemical, biological, and hybrid surface modification methods, aiming to enhance the TENG’s output performance by improving the contact area of porous dielectrics [66]. PPMs exhibit a range of excellent properties, including outstanding acoustic absorption and sound damping, superior thermal resistance, and good electromagnetic interference (EMI)-shielding characteristics [67,68]. Therefore, introducing these properties into PPMTENGs enables their development in emerging application areas, especially suitable for scenarios in harsh environmental conditions and with secondary objectives.

The pores within the dielectric play the role of air gaps in the operation cycle of TENGs. Creating air gaps in TENG devices using traditional methods is a challenging task (as shown in Figure 3b). In contrast, by using the pores inside the porous dielectric to replace air gaps, the design complexity can be reduced. Various design schemes have been proposed, including arc-shaped, spring-assisted, spacer-assisted, vibrational, and spherical designs, to provide the required air gaps between dielectrics. However, these designs often struggle to withstand numerous cycles. The changing morphology of the air gaps over time can have a negative impact on the output performance of the TENG. In fact, the porous structure’s framework acts as a spacer, allowing the TENG to exhibit the functions of traditional air gaps during regular contact and separation processes. In other words, these pores can be viewed as minute air gaps in micro-TENGs, and the collective of all these pores forms a series of TENG units. In this design, the requirement for external air gaps is obviated, since the porous dielectric incorporates a network of internal pores. When pressure is exerted and the porous dielectric undergoes compression, the pore size diminishes, leading to the generation of opposing charges and, thereby, a current in the external circuit. Once the porous dielectric is fully compressed and the external force is alleviated, the empty spaces within the porous membrane start to expand until they revert to their original state [69,70]. This results in a transfer of opposite charges in the external circuit (as shown in Figure 3c). Considering the primary characteristics of porous dielectrics and the energy-harvesting mechanisms in these materials, the following three subsections will discuss the main types of porous dielectrics, namely foam-based, aerogel-based, and fibrous medium-based dielectrics.

#### 3.1.1. Foam-Based Polymer Dielectrics

The preparation methods for porous polymer foam-based dielectrics mainly include template-assisted synthesis, foaming method, and laser-induced method. Template-assisted synthesis uses solid templates, such as salt crystals or sugar cubes, to coat the required materials on the template surface or inside. By curing or sintering and then removing the template, an interconnected porous polymer framework is formed, thus achieving precise control over the pore structure and diameter. The foaming method is a technique commonly used for producing porous-structured materials. By adding foaming agents, bubbles are produced, forming a porous structure. This method is simple, cost effective, and applicable to various materials [71,72]. The laser-induced method is an emerging technology for preparing porous foam structures, utilizing lasers to generate a thermal effect in PPMs, melting them locally, and producing bubbles to form a porous structure. This method is straightforward, highly precise, and can be used to design and create porous structures of different sizes and shapes, offering extensive application potential [42,73].

**Figure 4:**
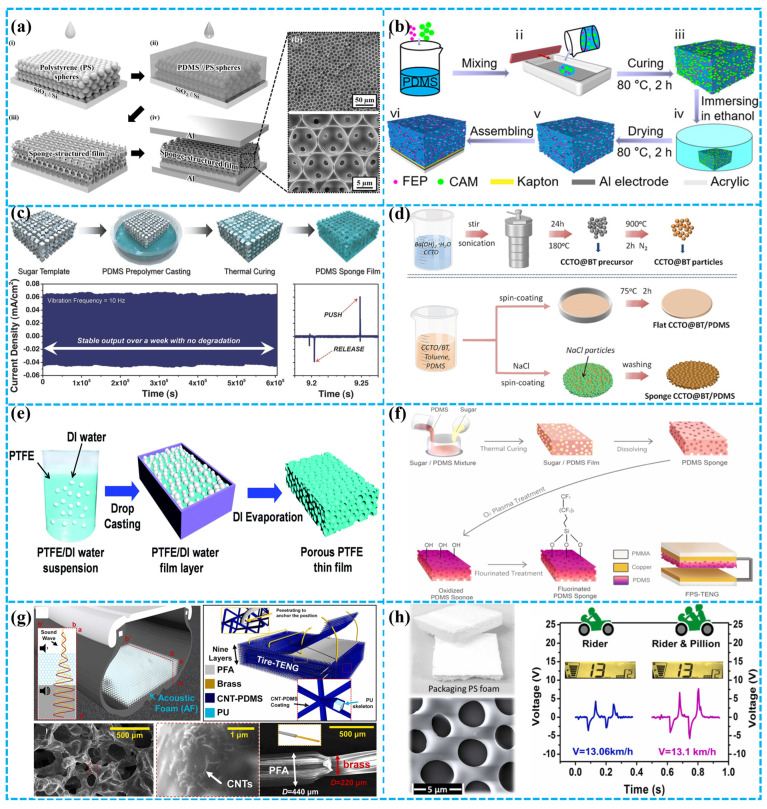
(**a**) STNG’s structure and fabrication. Reproduced with permission [74]. Copyright 2014, WILEY-VCH. (**b**) Porous PDMS and FB-TENG in single-electrode mode fabrication diagram. Reproduced with permission [75]. Copyright 2022, American Chemical Society. (**c**) Porous PDMS sponge fabrication using sugar particles. Reproduced with permission [76]. Copyright 2016, WILEY-VCH. (**d**) CCTO@BT particles fabrication. Preparing flat CCTO@BT/PDMS composite film and sponge. Reproduced with permission [77]. Copyright 2023, Elsevier B.V. (**e**) Porous PTFE thin film fabrication methods. A 3D image of the porous PTFE thin film from PTFE/DI water mixed with 50% DI water. Reproduced with permission [78]. Copyright 2017, The Royal Society of Chemistry. (**f**) Design of the FPS-TENG. Polymer sponge characterization with SEM images of large-hole sponge with 25 wt% PDMS. Reproduced with permission [79]. Copyright 2021, Elsevier Ltd. (**g**) Illustration of a tire with pristine AF. Tire-TENG schematic. SEM images of CNT-PDMS on a PU skeleton. Reproduced with permission [80]. Copyright 2022, Elsevier Ltd. (**h**) Photos of WPS material, recycled WPS film with Cu electrode, WPS film’s SEM image, and WPS-TENG photos. Reproduced with permission [81]. Copyright 2022, Elsevier Ltd.

To leverage the advantages of a porous structure, in 2014, and as shown in Figure 4a, Lee et al. used polystyrene microspheres as a template to obtain a porous sponge structure, which exhibited a 10-fold increase in power compared to a flat thin-film-based TENG (FTNG). This method demonstrates its potential in the development of high-performance TENGs [74]. Similarly, Kou et al. utilized citric acid as a solid template to obtain a flexible and breathable TENG that was employed in a range of applications, including head motion monitoring and bed exit alarm functions (Figure 4b) [75]. Kim et al. developed a manufacturing method for triboelectric sponges using a sugar cube template and 3D soft lithography (Figure 4c). The resulting sponge displayed remarkable properties such as superhydrophobicity and elasticity, rendering it applicable in various fields. With identical mechanical force, the power output of the sponge was 3 times higher than that of the control group (flat PDMS film). Lastly, they also achieved a high immunity for TES in extremely humid environments [76].

In a similar vein, as shown in Figure 4d, Lu and colleagues achieved the incorporation of high-dielectric CCTO@BT nanoparticles and pores within a PDMS structure via a feasible filling and removal process. They identified the significant influence of dielectric constant and porosity on the surface charge density and pore fraction of the dielectric in TENG design. As a result of their innovation, they achieved a 2.5-fold power enhancement in the TENG [77]. PTFE, ranking higher than PDMS in electronic attraction, offers significant potential for TENG development. As illustrated in Figure 4e, Wang’s team developed an S-TENG using a porous PTFE film, crafted with deionized water as a template. At optimal porosity (50% deionized water volume), the output voltage peaked at 5.1 V. This porous PTFE S-TENG delivered a voltage 1.8 times greater than its solid counterpart under the same conditions. Notably, when manually pressed, it generated 1.1 V, and inside a latex glove, this surged to 6.9 V, immediately lighting up five green LEDs without energy storage [78]. Peng and his team introduced a high-performance FPS-TENG crafted from a robust fluorinated polymer sponge, demonstrating unmatched electrical stability across various humidity levels. Owing to the sponge’s superior hydrophobic nature, the FPS-TENG resists moisture-related setbacks, ensuring consistent performance even after extensive wear (Figure 4f). At a relative humidity of 40%, this innovative FPS-TENG achieves an electrical surge of 181 V, 2.26 μA, and 52.5 μC m^−2^, marking a staggering 364% leap over traditional PPS and PPF-based TENGs [79]. In an innovative stride, Kim and his team introduced the Tire-TENG, a groundbreaking TENG integrated into a smart tire using acoustic foam (AF). Beyond its sound-damping capabilities, the AF acts as a potent energy collector, delivering both impressive energy output and noise mitigation. As depicted in Figure 4g, Tire-TENG’s prowess was substantiated through rigorous lab and Flat-Trac evaluations, proving its efficacy in powering smart sensor systems within the tire. With its dual capabilities of versatile energy capture and formidable environmental resistance, the Tire-TENG emerges as an optimal choice for continuously energizing smart tire sensor systems [80]. Nawaz and team pioneered a technique to harness polystyrene (PS) from discarded packaging, subsequently crafting a porous TENG showcased in Figure 4h. The device’s outstanding triboelectric charge density (~90 μC m^−2^) is a testament to the unique porosity introduced in the WPS film. Notably, the WPS-TENG showcased enduring stability, withstanding over 20,000 contact/separation cycles and a test period of 180 days. Leveraging this innovation, they also introduced a self-sustained speed sensor for road vehicles, underscoring the practical applications of the WPS-TENG [81].

From an overarching standpoint, foam processing offers a distinct advantage in meticulously engineering the size, distribution, and morphology of pores. Dielectrics grounded in foam technology stand at the forefront as prime contenders for seamless TENG architectures, with expansive void units showcasing dynamic, spring-like attributes, perfectly aligned for the TENG’s contact separation dynamics. Nonetheless, the presence of these pronounced voids inherently creates pronounced discontinuities in the polymer matrix, which could compromise its dielectric integrity. The realm also grapples with potential pitfalls such as fluctuating energy outputs, escalated manufacturing expenditures, diminished longevity due to consistent compressions, and variances in specimen parameters, potentially jeopardizing the consistency and replicability of performance outcomes.

#### 3.1.2. Aerogel-Based Dielectrics

Aerogels, with their ultralightweight, highly porous nature, boast a sophisticated three-dimensional nanonetworked architecture. Their intricate synthesis includes gelation, solvent extraction, and gas phase transitions [82]. Renowned for their minimal densities, vast surface areas, and superior thermal insulation, aerogels find applications in advanced insulation, sound attenuation, and as catalyst substrates. The production techniques for aerogels are continually optimized to suit diverse industry needs. Material chemistry and environmental conditions influence their unique pore structure, ranging from nanometers to microns, ensuring uniformity. This structure amplifies nanoconglomerations and surface-to-volume ratios. Being feather-light, aerogels are ideal for weight-sensitive applications. As dielectrics, they offer immense lightweight advantages and charge storage capacities, marking them as a preferred choice in innovative TENG designs.

**Figure 5:**
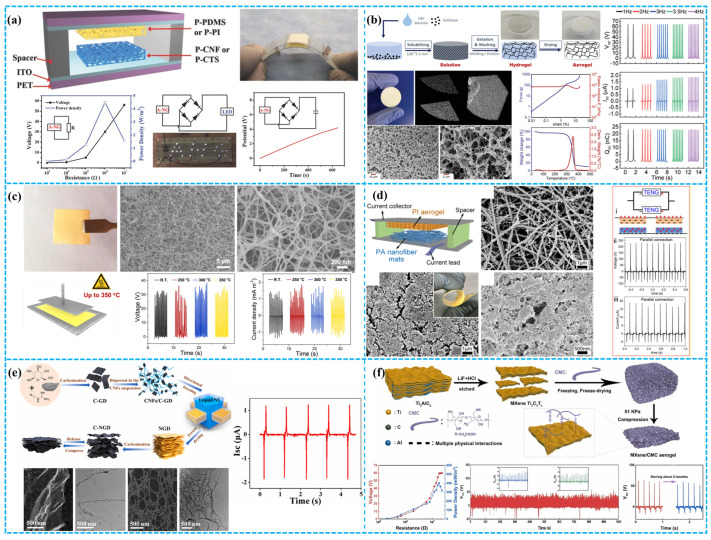
(**a**) A-NG: Porous aerogel film-based TENG with schematic and photo. Also, performance and stability of P-CTS/P-PI A-NG. Reproduced with permission [83]. Copyright 2018, WILEY-VCH. (**b**) Cellulose II structure: Fabrication, characterization, and performance under varying force and frequency. Reproduced with permission [84], Copyright 2020, WILEY-VCH. (**c**) PBOA/Al TENGs: Fabrication, SEM of compressed PBOA films, and schematic. Shows TENG performance under different temperatures. Reproduced with permission [85], Copyright 2019, Elsevier Ltd. (**d**) PA nanofiber mats and PI aerogel film TENG: Illustrates TENG setup and material morphology. Reproduced with permission [86]. Copyright 2018, American Chemical Society. (**e**) C-NGD: Details of preparation, SEM, and TEM images of nanosheets. Reproduced with permission [87], Copyright 2021, Elsevier Ltd. (**f**) MXene/CMC aerogel: Fabrication and evaluation of voltage/power density. Output stability of MXene/CMC2.5 aerogel-based TENG: Stability assessment at 2 Hz. V_oc_ curves of MXene/CMC aerogel TENG: Comparison before and after 5 months at room temperature. Reproduced with permission [67], Copyright 2022, Elsevier Ltd.

In 2018, Zheng’s team innovated a TENG (A-NGs) harnessing the capabilities of a cutting-edge polymer porous aerogel film. Capitalizing on an amplified contact area and innate electrostatic induction of the porous matrix, the A-NGs eclipsed the performance of traditional dense-film nanogenerators, D-NGs (Figure 5a). Employing a fusion of porous chitosan with a highly porous polyimide aerogel (at 92% porosity), this A-NG showcased a remarkable voltage of 60.6 V and a current of 7.7 µA, equating to a power density of 2.33 W m^−2^ [83]. Zhang and associates unveiled an innovative technique for engineering cellulose-based aerogel TENGs. Through a strategic dissolution–regeneration protocol, they achieved a cellulose aerogel endowed with a sophisticated 3D open-pore network, remarkable flexibility, pronounced porosity, and a significant surface area of 221.3 m^2^ g^−1^ (Figure 5b). TENGs harnessed from this cellulose II aerogel demonstrated superior mechanical responsiveness and elevated electrical performance [84].

As depicted in Figure 5c, Qian and team utilized polybenzobisoxazole aerogel (PBOAs) for the TENG as a resilient negative tribomaterial, withstanding up to 350 °C. Leveraging its pronounced porosity and superior specific surface area, the TENG recorded a maximum of 40 V open-circuit voltage, 2.9 mA m^−2^ current density, and 72 μC m^−2^ charge density. Remarkably, even at 350 °C, the TENG maintained a J_sc_ of 1.2 mA m^−2^ and a charge density of 32 μC m^−2^, underscoring its potential in high-temperature applications [85]. Leveraging porous polyamide (PA) nanofiber pads and polyimide aerogel films, Mi’s team unveiled an advanced TENG design (Figure 5d). Their study underscored the pivotal role of multilayered porous tribomaterials in optimizing TENG output. As PA layering progressed from single to sextuple, a marked surge in triboelectric efficiency was observed [86]. Simultaneously, multifunctional carbon aerogels have been integrated into TENG fabrication. Using a biomass-mediated strategy, Long’s team synthesized nitrogen-doped carbon aerogels (C-NGD) from a cost-effective, abundant blend of calcined glucose, dicyanamide nanoplatelets (C-GD), and cellulose nanofibers (CNFs). The synergy between C-GD and CNFs crafted a robust wavy lamellar architecture (Figure 5e). This carbon aerogel is not only apt for wearable piezoresistive sensors tracking bodily motions and biosignals but also showcases promise in supercapacitors and triboelectric nanogenerators [87]. As shown in Figure 5f, Cheng et al. developed an MXene-Ti_3_AlC_x_/carboxymethyl cellulose (MXene/CMC) aerogel for both mechanical energy harvesting and shielding against electromagnetic radiation, demonstrating a dual functionality [67].

**Figure 6:**
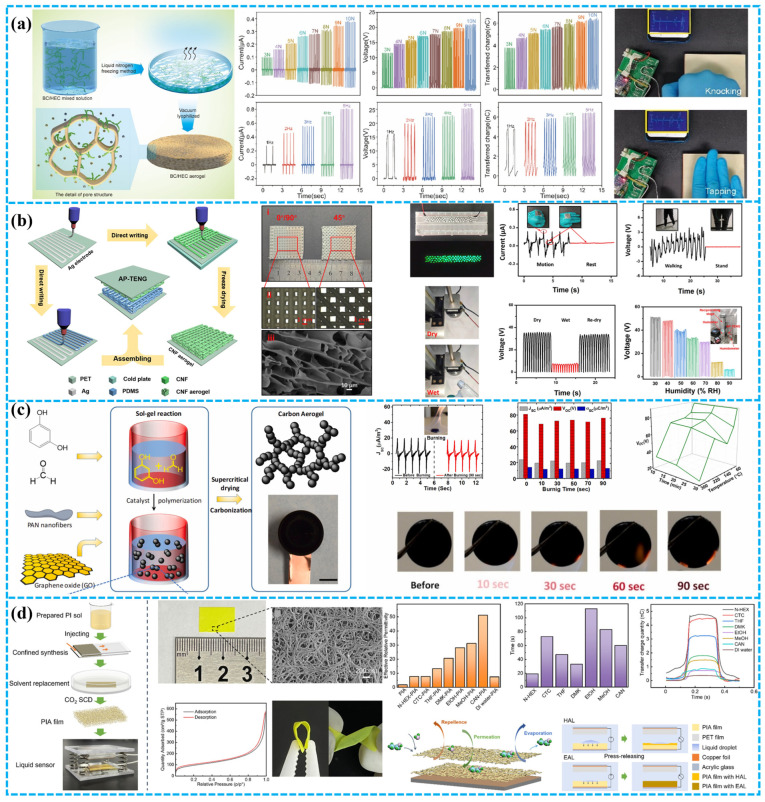
(**a**) BC/HEC aerogel preparation. Current, voltage, and charge of 20/80 aerogel TENG under 1 Hz frequency and varying external forces. Electrical signal on mobile screen upon tapping the smart panel. Reproduced with permission [88]. Copyright 2023, American Chemical Society. (**b**) Schematic AP-TENG fabrication. Photos (**i**) and corresponding optical microscope images (**ii**) of CNF triboelectric layer patterns at different tilt angles, with cross-sectional SEM image (**iii**) of printed aerogel structure. Reproduced with permission [89]. Copyright 2019, Elsevier Ltd. (**c**) Diverse applications of multifunctional AP-TENG. FRTENG creation steps. Flame resistance and self-extinguishing properties. Reproduced with permission [90]. Copyright 2019, Elsevier Ltd. (**d**) PIA film creation and liquid analyzer’s structural design. Mechanisms behind trace liquid analysis. Reproduced with permission [91]. Copyright 2023, American Chemical Society.

Luo et al. introduced a biocompatible porous TENG utilizing bacterial cellulose (BC) and hydroxyethyl cellulose (HEC) aerogels. The plentiful hydroxyl groups in BC and HEC molecules enable the formation of a three-dimensional network structure through hydrogen bonding, eliminating the need for additional cross-linking agents (Figure 6a). Through fine-tuning of the aerogel’s surface potential and pore structure, the output performance of the BC/HEC aerogel-based single-electrode TENG experiences a substantial boost. It outperforms pure BC aerogels with matching HEC content (80 wt%) by over 30 times and non-porous samples by over 4 times. This method delivers a biocompatible, cost-effective, and highly porous cellulose-based TENG with greatly enhanced output capabilities [88]. Qian et al. achieved a biocompatible cellulose-based TENG through advanced full printing. This AP-TENG, distinguished by a unique 3D micro/nanopatterned design, optimizes structural efficiency, increasing contact area, surface roughness, and mechanical resilience. This leads to heightened triboelectric response compared to traditional molded TENGs (Figure 6b). The layered micro/nano 3D structure of the AP-TENG delivers superior voltage output. This breakthrough provides a novel approach for crafting high-performance 3D TENGs with wide applicability in multifunctional electronics [89].

As aerogel synthesis advances, there is a renewed focus on improving their triboelectric performance. Common materials like polyimide (PI), polybenzimidazole, polyurethane, polyvinylidene fluoride (PVDF), polyethylene terephthalate (PET), and polyether ether ketone (PEEK) have shown significant progress in this area. Ahmed et al. developed a flame-retardant, self-extinguishing triboelectric nanogenerator (FRTENG) with controllable chemical and structural properties. This FRTENG, also functioning as a motion sensor and generator, utilizes resorcinol–formaldehyde aerogel’s excellent thermal properties. By incorporating polyacrylonitrile nanofibers and graphene oxide nanosheets, they significantly enhanced its electrical, mechanical, and triboelectric performance. This advancement enables the FRTENG to generate up to 80 V potential differences and achieve current densities of 25 µA/m^2^ (Figure 6c). Additionally, the FRTENG exhibits flame-retardant and self-extinguishing traits, marking a breakthrough in lifesaving wearable technology [90]. Zhou’s team created a highly porous 220 μm thick polyimide aerogel (PIA) film with an impressive 98.01% porosity. Using this material, they developed a liquid analyzer embedding a TENG structure (Figure 6d). This analyzer swiftly responds to tiny liquid volumes as small as 7 μL, owing to the PIA film’s mesoporous and highly porous yet robust nature [91]. By manipulating drying conditions and synthesis parameters, aerogel structures with diverse pore sizes and distributions can be attained. While aerogel-based dielectrics offer benefits like extreme light weight, superior insulation, and highly adjustable surface-area-to-volume ratios, they are hindered by brittleness and the challenge of thin-film fabrication. These factors can negatively impact the output of aerogel-based TENGs.

#### 3.1.3. Fiber Dielectric

**Figure 7:**
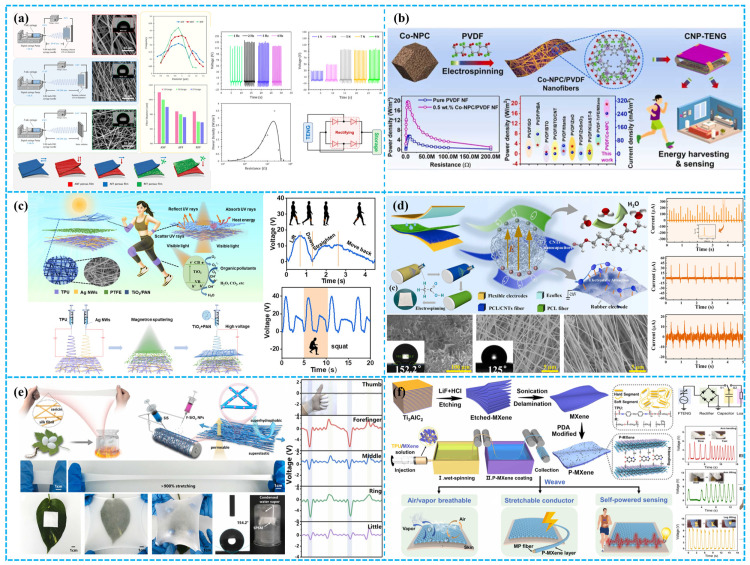
(**a**) Compares fabrication processes, SEM morphologies, and WCA of electrospinning samples, optimizing parameters. Reproduced with permission [93]. Copyright 2023, Elsevier Ltd. (**b**) Illustrates Co-NPC synthesis and PVDF composite NFs. Reproduced with permission [94], Copyright 2022, Elsevier Ltd. (**c**) Details of structure and working mechanism, showing leg actions and voltage signals during walking, and activities monitoring for squatting. Reproduced with permission [95]. Copyright 2021, American Chemical Society. (**d**) Presents BPF-TENG structure and biomechanical energy harvesting. Reproduced with permission [96], Copyright 2022, Elsevier Ltd. (**e**) Highlights SPSM fabrication and STENG arrays as self-powered sensors. Reproduced with permission [97], Copyright 2022, Elsevier Ltd. (**f**) Covers materials, structure, wet spinning, and smart textile functionality, with voltage signals for human motions. Reproduced with permission [98], Copyright 2023, Elsevier Ltd.

Fiber dielectrics, based on fibrous structures, are typically composed of polymeric materials. This imparts excellent flexibility and a large specific surface area. The pore size, crucial for charge storage, varies based on factors like fiber diameter and fabrication methods. Processing technologies include electrospinning, wet spinning, gel spinning, melt spinning, dry spinning, and centrifugal spinning [27,39,92]. Research-team-controlled factors like solution pumping rate and needle type regulate fiber size and distribution, allowing precise control over pore size and porosity in the prepared fiber pads. As shown in Figure 7a, Rastegardoost et al. developed a high-performance TENG using porous PVDF pads with enhanced dielectric properties and a unique dipole arrangement. Different single-layer electrospinning felts were produced by adjusting process parameters. The enhancement was even more significant in intelligent multilayer configurations, achieved by stacking electrospinning porous pads with oriented dipoles. The dielectric constant surpassed that of a single-layer electrospinning pad and matched the non-porous original PVDF film. Output voltages exceeded 130 V, with currents up to 12 μA—markedly superior to the non-porous original PVDF film and single-layer electrospinning pad [93]. Rahman et al. incorporated cobalt-based nano porous carbon (Co-NPC) derived from metal–organic frameworks into PVDF composite nanofibers (NFs) to enhance TENG performance in mechanical energy harvesting. Co-NPC, with large surface area and exceptional nanoscale porosity, significantly improved the β-phase formation and dielectric constant of PVDF composite NFs (Figure 7b). This led to a 4-fold increase in surface potential and a 9.5-fold increase in charge capture capability, resulting in a substantial boost in TENG efficiency [94]. Jiang et al. developed a self-powered, UV-protected, self-cleaning, and antibacterial triboelectric nanogenerator (TENG) based on Ag nanowires/TPU nanofibers and a TiO_2_@PAN network (Figure 7c). The TiO_2_ nanoparticles, evenly dispersed in PAN nanofibers, broaden solar absorption and enhance photocatalysis. With a micro–nanoporous structure, this TENG acts as a self-powered pedometer for monitoring human movement, promising diverse applications in human–machine interfaces [95]. Zhong et al. innovatively used asymmetrical dielectric manipulation, employing electrospinning to create a dual-layer PCL nanofiber felt. This material, distinguished by its large surface area, robust hydrophobicity, and high dielectric constant, functioned as an exceptional positive triboelectric material (Figure 7d). The BPF-TENG exhibited a 740% increase in transferred charge compared to PCL gel film-based devices, achieving 210 nC at 1 Hz. Remarkably, it could sustain long-term operation through human motion at 80% humidity [96]. In another study, as shown in Figure 7e, Li et al. achieved a milestone in material integration by developing a microfiber membrane that seamlessly combines hydrophobic elastic fibers with electrodes. Through synchronized electrospinning of styrene–isoprene–styrene (SIS) block copolymers and fluorinated SiO_2_ nanoparticles, they achieved a membrane with exceptional superelasticity, permeability, and superhydrophobicity. By integrating various conductors, including sputtered gold (Au) layers, they created a spectrum of elastic conductors and triboelectric active materials. These demonstrated optional benefits in terms of mechanical stability, electrical durability, triboelectric output, and permeability. This breakthrough gave rise to the development of self-powered sensors that are not only breathable but also capable of material identification and hand gesture monitoring [97].

In recent years, fiber dielectrics have gained prominence in the study of porous materials. Numerous reviews have specifically explored TENGs based on fibers and textiles, emphasizing the chosen fiber-processing techniques and material selections [92,99,100,101,102,103]. The TENGs incorporating fiber dielectrics have been categorized and studied. For instance, Kwak et al. systematically analyzed the organization of fibers within fibrous matrices. Their examination further elucidated the performance attributes of fiber-based configurations, encompassing wound, coaxial, folded, and elastic fiber architectures [100]. Hao et al. introduced a highly stretchable, conductive composite fiber formed by co-polymerizing surface-modified MXene (P-MXene) ink with wet-spun MXene/TPU fibers (MMP). MMP combines TPU’s mechanical flexibility with MXene’s conductivity, resulting in fibers with high conductivity (4.32 S cm^−1^), extensive strain tolerance (~675%), and strong mechanical properties (~3.76 MPa) (Figure 7f). These fibers, when woven with commercial ones, create fiber-based TENGs that convert mechanical energy to electricity, generating 20.1 V open-circuit voltage and 0.16 mW m^−2^ power density [98].

**Figure 8:**
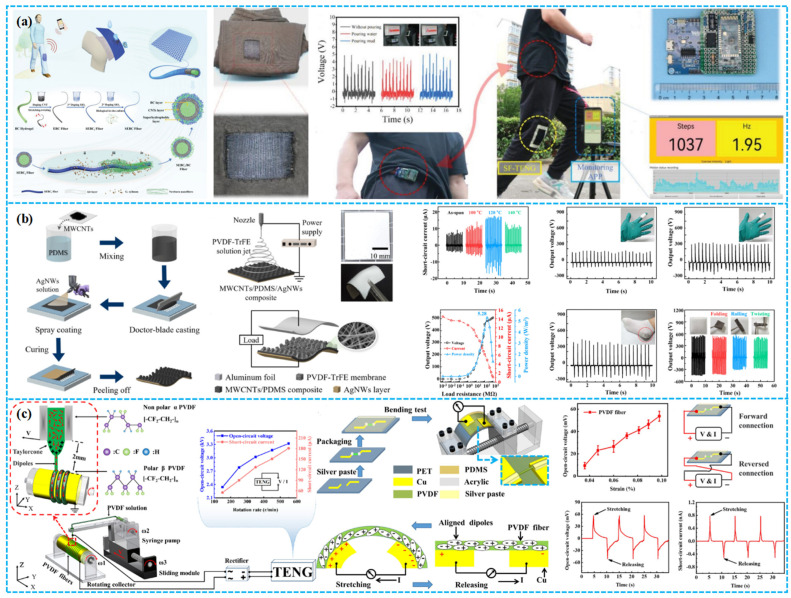
(**a**) Fabrication of SEBC fiber and SF-TENG. Overview of the intelligent clothing and a magnified image of the SF-TENG component. Performance results of the intelligent clothing and sports and health monitoring system. Reproduced with permission [105], Copyright 2023, WILEY-VCH. (**b**) Fabrication and structure of the TENG. Details of the TENG fabrication process and its structure. Real-time monitoring of human body motion. Output voltage, short-circuit current, and power density of the TENG annealed at 120 °C. Reproduced with permission [106], Copyright 2023, Elsevier Ltd. (**c**) Preparation of PVDF fibers via NFES system. Description of the PVDF fiber preparation process using the NFES system driven by a TENG. Workflow for testing the piezoelectric performance of single PVDF fibers, including forward and reversed connections for measuring open-circuit voltage and short-circuit current. Reproduced with permission [107]. Copyright 2023, American Chemical Society.

In addition to investigating two-dimensional (2D) and three-dimensional (3D) fiber structures, such as knitting, weaving, and braiding, this study also discusses and compares various techniques aimed at enhancing the triboelectric electrification of fibers. These techniques include coating, spinning, electroplating, and printing, as illustrated in Figure 8 [104]. For instance, Chen et al. have introduced an eco-friendly superhydrophobic fabric-based TENG (SF-TENG) composed of superhydrophobic conductive bacterial cellulose fibers (SEBC fibers) woven in a core–shell structure. SEBC fibers with this biofabricated core–shell structure exhibit outstanding conductivity, mechanical strength, biodegradability, and long-lasting superhydrophobicity (Figure 8a). This configuration achieves a maximum open-circuit voltage of 266.0 V, a short-circuit current of 5.9 μA, and an output power of 489.7 μW. SF-TENG successfully powers devices like stopwatches and calculators, offering a novel biomanufacturing strategy for core–shell superhydrophobic conductive fibers [105].

Despite the introduction of various methods for producing nanofibers, including techniques like self-assembly and phase separation, electrospinning remains the dominant approach in this research field. Its widespread use is attributed to its ability to control fiber diameter and microstructure, cost effectiveness, and the availability of a diverse range of materials [108,109]. Furthermore, over 55% of these studies opt for fluorinated polymers as the material for nanofiber production. These polymers, composed of fluorine and hydrogen atoms, exhibit dipole moments in different configurations, resulting in superior triboelectric electrification performance. Ge et al. provided an overview of the factors influencing the preparation and formation of electrospinning fibers, as well as their advantages as triboelectric electrodes for TENGs [110]. Recently, Lee et al. fabricated a highly flexible TENG by directly electrospinning polyvinylidene fluoride–trifluoroethylene (PVDF-TrFE) nanofiber membranes onto a multiwalled carbon nanotube (MWCNT)/polydimethylsiloxane (PDMS)/silver nanowire (Ag NW) composite electrode (Figure 8b). The electrospun PVDF-TrFE nanofiber membrane, with its unique crystal structure, enhances the TENG’s output performance and ensures stable electricity generation under various conditions [106]. Guo et al. utilized a TENG-powered near-field electrospinning (NFES) system to craft PVDF fibers with precision. They achieved controlled deposition on a rotating drum electrode by short-distance (2 mm) continuous injection of PVDF precursor solution from a moving needle, eliminating the need for extra polarization and stretching. Key parameters like PVDF concentration, needle diameter, TENG pulse DC voltage, flow rate, and drum speed were systematically optimized for the desired β-phase fraction. At a 0.5 Hz frequency, the PVDF single fiber device yielded 6.1 mV voltage and a maximum power of 3.52 pW with an optimal load resistance of 10.6 MΩ (Figure 8c). This cost-effective TENG-driven NFES approach offers highly controllable PVDF fibers suitable for precision micro/nanodevices and wearable components [107].

Li et al. provided an extensive review of recent progress in electrospinning nanofibers used for triboelectric-based energy generation and functional conductors. They covered topics such as working mechanisms, fabrication strategies, geometric control, functional integration, and performance enhancements. Additionally, the review emphasized the advantages of nanofiber-based TENG devices across different application areas, highlighting their potential for harvesting high-entropy energy from the environment [39]. In summary, fiber dielectrics offer advantages such as strain compatibility, breathable structures, and versatile control over dielectric thickness—a key TENG parameter—making them potential candidates for diverse applications. However, they have limitations including limited mechanical strength, reduced structural integrity under cyclic loading, significant interface challenges, sensitivity to environmental conditions, and a need for improved chemical stability.

### 3.2. Porous Electrode Materials

**Figure 9:**
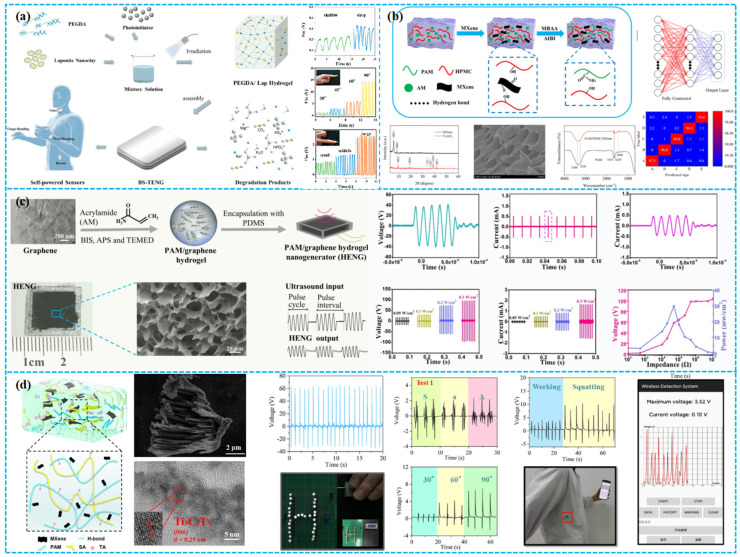
(**a**) Preparation schematic of BS-TENGs using PEGDA/Lap hydrogel for physiological signal monitoring. Reproduced with permission [114]. Copyright 2023, American Chemical Society. (**b**) Synthesis outline of PAM/HPMC/MXene hydrogel with a confusion matrix for 1D-CNN prediction. Reproduced with permission [115]. Copyright 2023, American Chemical Society. (**c**) Visualization of ultrasound-activated electrical stimulation of vagus nerves via implantable HENG, with corresponding ultrasound response. Reproduced with permission [116], Copyright 2021, Elsevier Ltd. (**d**) Hydrogel structural diagram with repeatable voltage signals highlighting handwriting detail discernment. Reproduced with permission [117]. Copyright 2023, American Chemical Society.

Porous electrode materials are pivotal in TENG for efficient energy collection and conversion. Critical considerations for these materials include conductivity, surface area, pore architecture, and mechanical and chemical robustness [111,112]. The evolution to an all-porous TENG design offers superior breathability, flexibility, and adaptability, positioning it ideally for advancements in flexible electronics and wearable tech. This adaptability ensures consistent performance across diverse environments and dynamic conditions [113]. Li’s team adeptly engineered a PEGDA/Lap nanocomposite hydrogel conductor from biocompatible polyethylene glycol diacrylate and lithium laponite, leading to the creation of a pioneering biodegradable single-electrode triboelectric nanogenerator (BS-TENG) (Figure 9a) [114]. Fabricating high-conductivity porous electrodes presents a significant challenge due to the inherent tension between conductivity and porosity. Elevated conductivity often diminishes porosity, given the limited surface area of highly conductive materials. This demands a nuanced balance between the two. Importantly, such enhancements can pose a risk to mechanical resilience, potentially compromising electrode longevity. In TENG design, the highlighted challenges can precipitate notable energy losses, compromising output efficiency. Beyond the electrode’s conductivity and porosity, a pivotal concern is achieving robust adhesion between the electrode and dielectric for optimal charge transfer. An uptick in pore density and size can diminish this electrode–dielectric contact area, undermining adherence. Such a decline can detrimentally influence the TENG’s long-term performance. Consequently, in the electrode fabrication process, it is essential to judiciously balance conductivity, porosity, and effective contact interfacing.

Beyond electrode conductivity and porosity, commercialization of porous electrodes grapples with two salient barriers: prohibitive production costs and diminished durability. This underscores the imperative for intensified research focus. Fabricating high-conductivity porous electrodes involves intricate methodologies, necessitating precise control over processes and parameters. While multiple methods exist, balancing effective electrode–dielectric contact with optimal conductivity and porosity remains a cardinal concern. Key conductive materials feature low-dimensional nanostructures, including nanowires, nanoparticles, and nanosheets, supplemented by conductive polymers like PANI and PEDOT. An alternate tactic entails the deposition of conductive coatings on insulating substrates, utilizing materials like carbonaceous fillers, liquid metals, and electrolytes. Such components are foundational in crafting porous electrodes. Another pivotal avenue is the exploration of porous electrode fabrication techniques. Core methodologies include coating, electrospinning, metal plating, and printing. Utilizing these advanced techniques, researchers have synthesized diverse porous electrodes, such as aerogels, fibrous media, and foams, exhibiting properties like high transparency, stretchability, self-healing, and freeze resistance. Such advancements fortify the trajectory of porous electrodes, underscoring their potential for broad applications. The pronounced wettability of hydrogels markedly facilitates ionic conductivity, mitigating carrier constraints. In TENG designs, a common approach integrates moisture-retentive hydrogels within highly extensible elastomers. Utilizing sol–gel processes, factors such as pressure, temperature, and cross-linking density can be finely tuned, optimizing pore geometry in hydrogels and thus enhancing electrode efficacy. This innovation heralds a significant leap in TENG technology [118].

Li and colleagues devised a composite hydrogel comprising polyacrylamide, hydroxypropyl methylcellulose, and MXene (Ti_3_C_2_Tx) nanosheets. Through hydrogen bonding, the hydrogel establishes a robust double-helix structure, exhibiting attributes like superior strength, tensile performance, conductivity, and strain sensitivity (Figure 9b). Capitalizing on these properties, they constructed a flexible multifunctional TENG that can harness biomechanical energy, achieving a conversion of 183 V with a peak power density of 78.3 mW/m^2^ [115]. Panwar et al. synthesized a high-performance CMCh-CMC-PDA hydrogel by integrating carboxymethyl chitosan (CMCh) and CMC-dialdehyde-polydopamine (CMC-D-PDA) through both physical and covalent interactions. The pre-formation of CMC-D-PDA involved two key steps: oxidation of CMC to introduce aldehyde groups and subsequent dopamine polymerization. Similarly, actions on CMCh formed reversible dynamic imine bonds, yielding a hydrogel with outstanding properties [119]. Uniform dispersion of carbon-based materials in a hydrogel is a known challenge. Yet, Chen et al. ingeniously addressed this by integrating graphene dispersion with the hydrogel precursor, as depicted in Figure 9c. Introducing 5 wt% graphene into the PAM hydrogel created a conductive three-dimensional network, facilitating charge transfer and current collection [116]. While hydrogels offer excellent flexibility and stretchability for wearable electronics, their limited mechanical strength poses challenges for long-term stability. To address this, researchers have focused on developing self-healing hydrogels and resultant H-TENGs. Zhang et al. introduced an ionic hydrogel comprising polypropylene amine (PAM), tannic acid (TA), sodium alginate (SA), and MXene (PTSM). The hydrogel, fortified by numerous weak hydrogen bonds, demonstrated remarkable stretchability (strain > 4600%), adhesion, and self-repair capabilities. Encasing PTSM hydrogel with Ecoflex yielded the PTSM TENG, achieving an output power density of 54.24 mW/m^2^ (Figure 9d). This technology was integrated into a glove-based human–machine interaction (HMI) system [117]. Due to their outstanding strain compatibility, hydrogels are considered a potential choice for lateral sliding TENG applications. However, they require encapsulation, which to some extent limits their widespread use.

**Figure 10:**
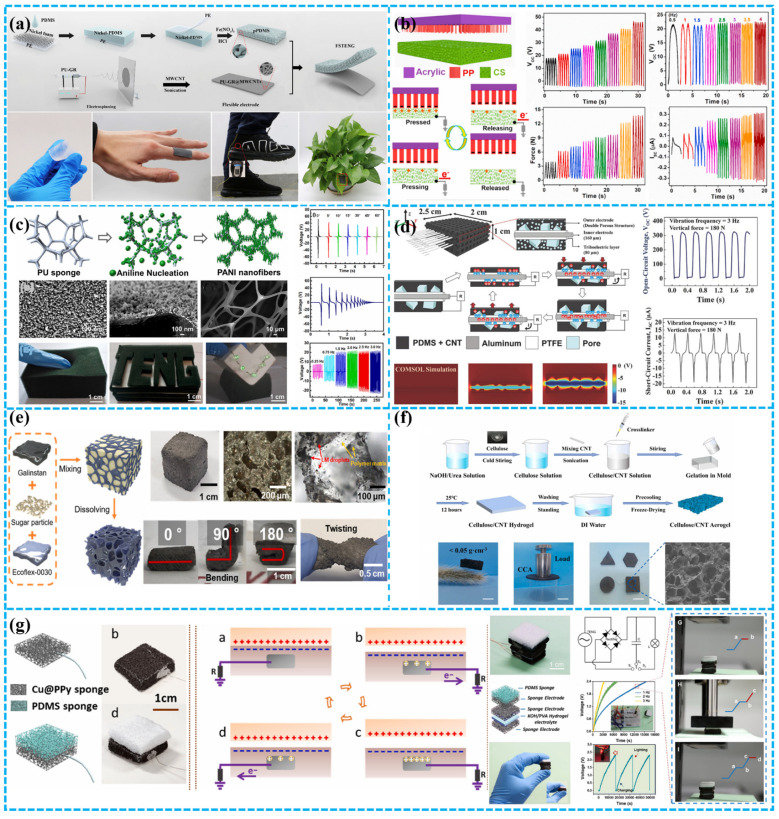
(**a**) Illustration of FSTENG fabrication process. Digital photo of resulting pPDMS film. Reproduced with permission [120]. Copyright 2019, Elsevier Ltd. (**b**) Electrical characterization of CS-based TENG in vertical contact separation mode. Reproduced with permission [121], Copyright 2021, Elsevier Ltd. (**c**) Illustration and SEM images of conductive elastic sponge preparation using dilute chemical polymerization. Schematic of ES-TENG. Reproduced with permission [122]. Copyright 2020, Elsevier Ltd. (**d**) Structure and operation of PCP-TENG. Electrical output under 180 N vertical force and 3 Hz frequency. Reproduced with permission [123], Copyright 2018, WILEY-VCH. (**e**) Fabrication process and photo of LMS. Reproduced with permission [124], Copyright 2021, Elsevier Ltd. (**f**) Illustration of CCA fabrication. Reproduced with permission [125], Copyright 2022, Elsevier Ltd. (**g**) Sponge-based TENG fabrication and corresponding output parameters. Reproduced with permission [126], Copyright 2019, Elsevier Ltd.

In addition to hydrogels, research has also been conducted on other porous electrode materials. Li et al. innovated a fully stretchable triboelectric nanogenerator (FSTENG) comprising electrospinning electrodes and a porous PDMS triboelectric layer with nickel foam structure (Figure 10a). The FSTENG achieves an impressive 92 V output voltage, surpassing traditional TENGs based on flat PDMS films by an order of magnitude [120]. Porous foam electrodes have been shown to enhance TENG performance. For example, Cui et al. developed a dual-mode TENG with a spongy electrode-brush structure for mechanical energy harvesting and self-powered trajectory tracking. The conductive sponge (CS) electrode, created through chemical and electroplating processes, features a flexible, elastic, porous, and large-surface-area network structure (Figure 10b). This CS-based TENG exhibits potential for self-powered sensing, excelling in both contact separation and sliding modes with excellent electrical performance and environmental adaptability. A 4 × 4 CS unit-based trajectory tracking matrix demonstrated outstanding real-time monitoring and comprehensive trajectory recording. This work carries substantial implications for the practical implementation of TENGs in future intelligent systems [121]. As shown in Figure 10c, Liu et al. have developed a TENG using conductive elastic sponges for efficient collection of random mechanical energy and ammonia sensing. The TENG is based on conductive sponge electrodes with large surface area, flexibility, and elasticity. It effectively harvests mechanical energy from random motion and vibration and detects ammonia. The TENG showed good sensitivity and stability in ammonia-sensing experiments and has potential for environmental monitoring and gas-sensing applications. Conductive elastic sponges and TENGs have potential to develop into a convenient self-powered source for collecting random mechanical energy and rapid response self-powered NH_3_ sensors. This work emphasizes the effectiveness of TENGs based on conductive elastic sponges in energy-harvesting and sensing applications [122].

In another study, Kim et al. demonstrated a TENG using the inner sponge surface as the contact interface. They prepared a porous conductive polymer (PCP) by adding multiwalled carbon nanotubes (MWCNTs) to elastic PDMS (Figure 10d). A PTFE-coated aluminum wire (PTFE Al wire) was inserted into the PCP, greatly increasing the effective contact area. This resulted in a high-volume charge density of 60 mC m^−3^. Degradation was only observed after 7 million contact separation cycles. The PCP-TENG efficiently harvested mechanical energy from various vibration directions and amplitudes, successfully extracting energy from tire deformation [123]. Similarly, Park et al. developed a highly flexible liquid metal embedded sponge-type TENG (LMST). It achieves impressive bending (up to 180°) and stretching (up to 300%). With dimensions of 1.5 cm × 1.5 cm × 1.5 cm, the LMST generated 188 nA of I_sc_ and 24 V of V_oc_. It achieved a power density of 2.48 W/m^2^ without traditional electrode manufacturing. Additional wires can further increase output. Three-dimensional printing allows for easy customization (Figure 10e). The LMST’s unique structure enables self-powered sensing (pressure, rotational ball direction, real-time motor fault detection), making it promising for battery-less sensors in diverse environments [124].

Aerogels have garnered significant attention as another category of porous electrodes in recent years. Wang et al. recently introduced a biodegradable, moisture-resistant cellulose carbon nanotube aerogel triboelectric nanogenerator (CCA-TENG) with a simplified structure. Serving as both triboelectric layer and electrode, CCA exhibits enhanced dielectric properties and a 3D porous structure, resulting in superior output performance compared to other cellulose-based TENGs (Figure 10f). Significantly, CCA-TENG can rapidly degrade in cellulase, enabling the recovery of CNTs for the reparation of CCA-TENGs, achieving 91.04% of the original TENG output performance [125]. Li et al. introduced a Cu-doped PDMS sponge as flexible and durable electrodes for TENGs and supercapacitors. They achieved tunable TENG performance using a Cu sponge loaded with polypyrrole (PPy). This material-driven approach enhances understanding of triboelectric generation (Figure 10g). Additionally, the Cu@PPy sponge improved the stability and performance of supercapacitors, making them suitable for wearable energy storage. This research opens new possibilities for multifunctional power sources and wearable electronics [126]. In another study, Peng et al. created a breathable, biodegradable electronic skin with antibacterial properties. Using a fully porous nanofiber TENG incorporating Ag NW within a structure of polylactic-co-glycolic acid (PLGA) and polyvinyl alcohol (PVA), the skin enables efficient heat and moisture transfer. It achieves a peak power density of 130 mW m^−2^ and a voltage response pressure sensitivity of 0.011 kPa^−1^, allowing for real-time, non-invasive, and self-powered physiological signal acquisition [127].

### 3.3. Porous Structure Design

In designing porous TENG devices, alongside the use of porous materials as dielectrics and electrodes, an alternative approach involves the meticulous design of porous structures, encompassing fabric and yarn-based configurations. Unlike pre-formed porous materials, the establishment of porous structures occurs during the device assembly phase, typically at a macroscopic scale [128,129]. Precise control over pore size and distribution is achieved by manipulating the layout of fibers, yarns, and ribbons, thereby enhancing uniform charge distribution. Fabric and yarn-based structures confer distinctive advantages in TENG devices, notably amplifying power generation efficiency, accommodating diverse operational environments, and offering superior flexibility and controlled fabrication processes [130]. This technology exhibits significant potential in advancing and applying TENG technology.

#### 3.3.1. Textile-Based TENG Structures

The first category of porous structure design, utilizing textiles as a foundation, creates macroscopic voids crucial for air gaps in TENG functionality. Advanced manufacturing techniques, such as weaving, arranging, and stacking 1D (fibers) or 2D (ribbons) dielectrics and electrodes, enable diverse porous TENG textures. This design simplifies traditional elastic spacer structures and propels the advancement of TENG technology [99,101,131]. These achievements invigorate the textile industry and offer unprecedented opportunities in energy conversion. The swift progress of generative textiles holds immense potential, not only in TENG technology but also in applications like wearable electronics and flexible sensors.

**Figure 11:**
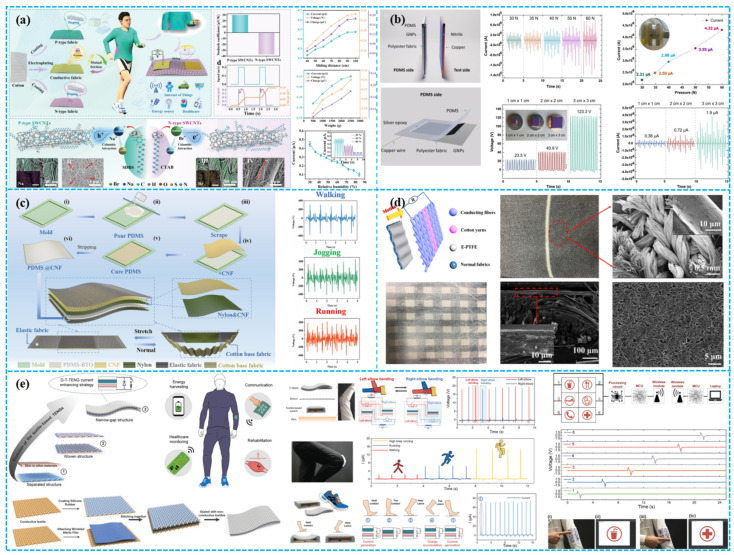
(**a**) AFDC-TENG preparation, application scenarios, and structures. Electrical output of p-type AFDC-TENG with different factors. Reproduced with permission [132], Copyright 2023, WILEY-VCH. (**b**) Schematic of PDMS-nitrile TENG and the fixed PDMS side. TENG performance under different forces and areas. Reproduced with permission [133], Copyright 2023, Elsevier Ltd. (**c**) Design and fabrication of AesF-TENG. Output voltage of wearable AesF-TENG-S under different motion statuses. Reproduced with permission [134], Copyright 2023, Elsevier B.V. (**d**) Structure of laminated fabrics and TENG textiles. Reproduced with permission [135], Copyright 2019, Elsevier Ltd. (**e**) Evolution of T-TENGs. Energy harvesting from diverse body motions. Wearable wireless communication board. Reproduced with permission [136], Copyright 2019, WILEY-VCH.

Lv et al. recently fabricated p-type and n-type fabrics with semiconductor properties by doping single-walled carbon nanotubes (SWCNTs) with organic molecules. These fabrics were used to develop three all-fabric direct current triboelectric nanogenerators (AFDC-TENGs) based on the triboelectric voltage effect (Figure 11a). By reciprocatingly rubbing them against a nickel-coated conductive fabric, they achieved high flexibility, comfort, and stable DC output. These AFDC-TENGs demonstrated outstanding characteristics, making them suitable for wearable applications [132]. In another study, Sadanandan et al. created a wearable fabric-based TENG, named the PDMS-Nylon TENG, by ultrasonically coating graphene nanosheets onto a polyester fabric for electrodes (Figure 11b). They applied PDMS polymer as the dielectric layer on one side of the TENG. This device achieves a power density of up to 0.3 W/m^2^. It holds promise for integration as an energy-harvesting component in wearables and as a pressure sensor in self-powered smart textiles [133]. Gao et al. developed an asymmetric elastic fabric-based triboelectric nanogenerator (AesF-TENG) that harmonizes with human motion, facilitating easy triggering of the contact separation process during diverse motion modes (Figure 11c). The AesF-TENG achieves a peak power density of 1067 mW m^−1^. Moreover, it serves as a self-powered wearable sensor for wireless monitoring of human motion [134].

Increasing focus on developing PPMTENG integrated textiles for scalable applications has laid a strong foundation for commercial viability through material selection, process optimization, and practical implementation. Huang et al. presented a design and production strategy for high-performance TENG textiles, emphasizing industrial-scale production. Among various designs, TENG fabrics with surface loops and higher static density demonstrated optimal performance. TENG textiles featuring a grating structure facilitated charge, current, and energy accumulation (Figure 11d). With pores of about 100 µm, their design achieved an output voltage of 800 V, rivaling TENGs improved through complex processes [135]. He et al. proposed an efficient strategy to boost triboelectric output by integrating narrow-gap TENG textiles with high-voltage diodes and textile-based switches (Figure 11e). The resulting diode-amplified T-TENG (D-T-TENG) achieved a 25-fold increase in closed-loop current compared to standalone T-TENG, with a 4-fold improvement in capacitor charging rate. The D-T-TENG was employed for energy harvesting from walking, powering a Bluetooth module in clothing, and for environmental sensing. This work advances wearable textile-based healthcare applications [136].

#### 3.3.2. Yarn-Based TENG Structures

Yarn-based TENGs, inspired by traditional textile craftsmanship, efficiently convert energy through the skillful combination of fine-fiber materials. This structure involves weaving fine fibers into yarn, creating microscale pores and structures that facilitate the triboelectric effect of TENGs. The fabrication process employs advanced techniques like spinning and weaving, ensuring the precision and stability of the structure, ultimately leading to outstanding energy-harvesting efficiency [137,138]. Designing yarn-based TENGs presents a critical challenge: maintaining the required gap during contact and separation operations. This entails considering key factors in the structural design and fabrication process. Firstly, selecting fiber materials with suitable mechanical and electrical properties is crucial. These materials should offer ample flexibility and strength to prevent breakage or deformation during contact and separation. Secondly, integration of yarn-based TENGs into a device or system necessitates ensuring the stability and controllability of the gap in practical applications. Lastly, designing appropriate contact and separation mechanisms is essential for reliably generating the required gap during operation, taking factors like yarn bending radius and contact angle into account [139].

**Figure 12:**
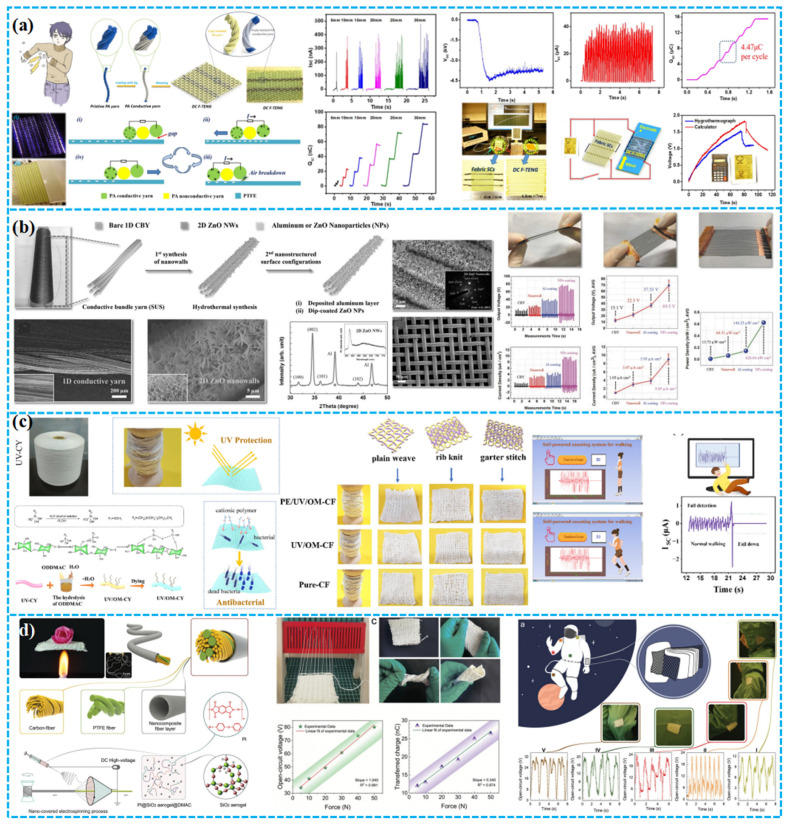
(**a**) Overview, creation, and functionality of the DC F-TENG. Examination of test variables on output and showcases of its energy-capturing capability. Utility scenarios and electric outcomes of the DC F-TENG. Reproduced with permission [140], Copyright 2020, American Chemical Society. (**b**) Blueprint and assembly outline for the 1D CBY-TENGs. Electrical results of the 1D CBY-TENGs. Reproduced with permission [141], Copyright 2017, WILEY-VCH. (**c**) Formation and analysis of UV/OM-CY. Images of yarn varieties aligned with three textile configurations. Display captures from the live motion-tracking system and graphical representation of fall detection. Reproduced with permission [142], Copyright 2023, Elsevier B.V. (**d**) Crafting and configuration of the nanoaerogel-enveloped triboelectric yarn. Blueprint and electric evaluations of Y-TENG under assorted stimuli. Presentation of an autonomous motion-trait-tracking setup consolidated with protective attire and the Y-TENGs. Reproduced with permission [143], Copyright 2022, WILEY-VCH.

Most yarn-based TENGs in advanced designs function in single-electrode mode. Due to material interactions in these configurations, yarn-based TENG woven pads exhibit greater thickness compared to their textile- and fiber-based counterparts. As a result, they present larger interstitial gaps. While fabric-based designs have been leveraged for porous TENG pads, yarn-based approaches also merit substantial attention. For example, Chen et al. devised a DC F-TENG using polyamide and nylon as warp and weft, respectively. In their systematic study on 16 fabric types, structural parameters and testing conditions were assessed for their impact on the DC F-TENG output (Figure 12a). Notably, their compact model (1.5 cm × 3.5 cm) illuminated 416 LEDs, whereas the larger version (6.8 cm × 7 cm) yielded V_oc_, I_sc_, and Q_sc_ of about 4500 V, 40 μA, and 4.47 μC per cycle. This work underscores the potential of textile-based TENGs to capture direct current energy via air breakdown, marking their viability as a power source [140]. Ko et al. introduced a 1D CBY-TENG, crafted from 1D conductive bundle yarn and 2D conductive fabric arrays, noted for its superior flexibility, durability, and wearability. Nanostructuring enhanced its triboelectric and charge induction capabilities (Figure 12b). With current and power densities of 17.16 µA cm^−2^ and 1.13 mW cm^−2^, respectively, its efficacy as a power source is underscored [141]. Cheng et al. developed a PE/UV/OM-CY TENG showcasing antimicrobial, UV-protective, and radiative cooling attributes. Using conductive stainless steel (SS) wire as core–shell yarn electrodes, it combines UV-resistant, antimicrobial cotton yarn (UV/OM-CY) with polyethylene (PE) yarn tightly coiled around the SS core (Figure 12c). This design holds profound potential for wearable tech textiles, antimicrobial applications, smart sportswear, and self-sustained systems [142]. In addition, Xing et al. devised a flexible, high-temperature-resistant Y-TENG. Through electrospinning and ancient twisting, they produced multilayer silica aerogel nanocoated triboelectric yarn, exhibiting outstanding high-temperature and triboelectric capabilities (Figure 12d). Operating between 25 and 400 °C, it yields a charge density of 30 nC cm^−2^, with a resistance of 180 MΩ, a power peak of 0.17 mW, and a sub-15 ms response time. The design further offers flame resistance, lightweight attributes, and cost efficiency [143].

## 4. Application of Porous TENG

The TENG, characterized by its distinctive high porosity, expansive surface area, and inherent compressibility, serves as a cornerstone in the fields of energy harvesting and self-powered sensing. Its adaptability has further enabled advancements in intelligent wearable technologies and has bridged innovative integrations within human–machine interfaces.

### 4.1. Energy Harvesting

Since its inception, a paramount objective of TENGs has been to harness energy from both the environment and living organisms. The advent of PPMTENGs heralds a new era, paving the way for sustainable and eco-friendly energy solutions.

**Figure 13:**
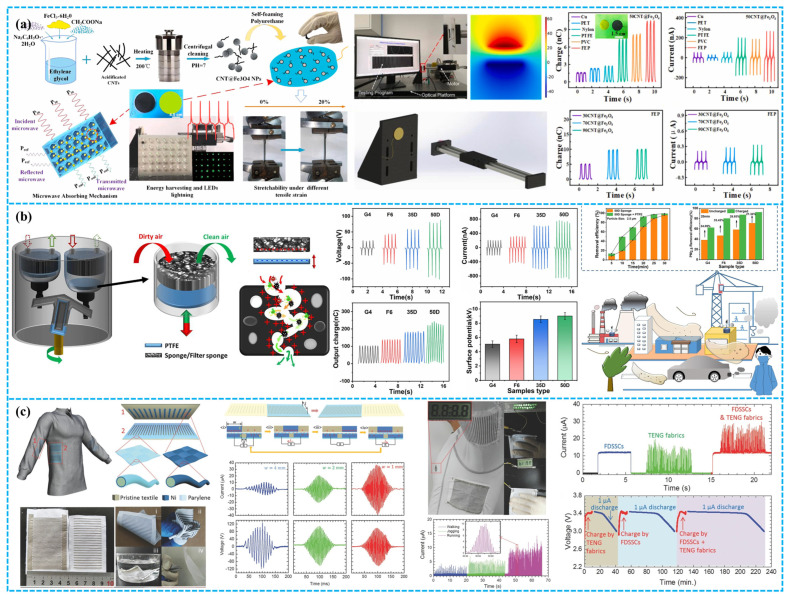
(**a**) Preparation procedure of the CNTs@Fe_3_O_4_/PU CF; microwave-absorbing mechanism. The potential distribution simulated by COMSOL software using the finite-element method. Short-circuit current of the CF-TENG with different triboelectric materials. The transferred charges and short-circuit current of the CF-TENG with different CNT contents. Reproduced with permission [144], Copyright 2021, Elsevier Ltd. (**b**) Conceptual schematic diagram of triboelectric air filtration system. The working principle and performance of TENG. TAFS filtration efficiency and application. Reproduced with permission [145], Copyright 2022, Elsevier B.V. (**c**) Fabrication of TENG fabrics. The scheme of a power-textile with a pair of TENG fabrics consisting of a slider fabric. The working mechanism of TENG fabrics. The demonstration of TENG fabrics. Reproduced with permission [146], Copyright 2016, WILEY-VCH.

In modern times, the convergence of clean energy capture and electromagnetic shielding in one material has garnered significant attention. For example, as shown in Figure 13a, Zheng and colleagues innovated a stretchable CF-TENG using self-foaming polyurethane (PU) and tadpole-like materials, integrating tunable microwave absorption (MA). The employment of CNTs@Fe_3_O_4_ as conductive filler and its uniquely shaped nanoparticles enhanced both triboelectric output and MA efficacy. Consequently, the CF-TENG boasts a peak output of 147.9 µW (density: 1.3 µW/cm^2^) and a 1.1 µA current, adeptly harvesting mechanical energy to illuminate 35 commercial LEDs. Merging electromagnetic shielding with mechanical energy scavenging, this CF-TENG promises transformative applications in wearable tech and future energy systems in challenging unseen environments [144]. Li et al. introduced a triboelectric air filtration system (TAFS) leveraging TENG technology (Figure 13b). This TAFS actively filters outdoor particulates, notably harmful PM2.5, by integrating an industrial filter medium with PTFE, thus boosting filtration efficiency. Efficacy for PM1.0 and PM2.5 interception is enhanced by 20–40%, with the triboelectric-induced positive charge also augmenting the capture of Staphylococcus aureus. The TENG-based self-suction air filtration system, encompassing an air pump, demonstrated 99% PM2.5 removal in a smoke-saturated space for 30 min. The wind cup design ensures efficient wind energy capture, resulting in self-sustainability, zero emissions, and heightened environmental protection. This self-powered TAFS offers a groundbreaking approach for air purification in environments like construction sites, factories, and sandstorms [145]. Pu et al. devised a grating-structured TENG fabric, integrated with fiber-shaped dye-sensitized solar cells (FDSSCs), to form a comprehensive textile-based energy harvester. The fabric, crafted from nickel-coated polyethylene (Ni-cloth) and parylene-coated nickel (P-Ni-cloth) strips, transduces low-frequency human motion into high-frequency electrical outputs. This union of FDSSCs and TENG fabric efficiently harnesses energy from solar and human kinetics [146].

### 4.2. Intelligent Wearable Devices

**Figure 14:**
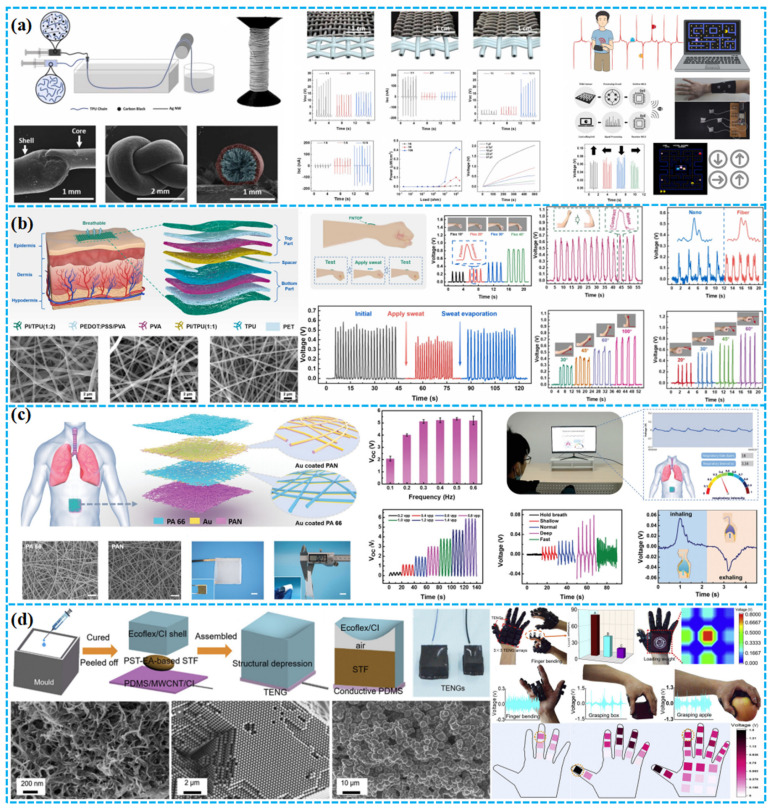
(**a**) Schematic representation of core–shell fiber wet spinning. Fabrics showcasing 1/1, 2/1, and 3/1 warp-to-weft ratios. Conceptual visualization of an IoT wristband in action. Reproduced with permission [147], Copyright 2023, Elsevier Ltd. (**b**) Depictions of the FNTOP on skin; on-skin patch placement on the wrist and its output testing with synthetic sweat. Voltage response of FNTOP to arm movement. Reproduced with permission [148], Copyright 2022, Elsevier Ltd. (**c**) Blueprint and mechanism of the TENG-powered SANES; system for respiratory assessment. Reproduced with permission [149], Copyright 2021, WILEY-VCH. (**d**) TENG device fabrication steps; hand array based on TENG and its gesture detection capability. Reproduced with permission [150], Copyright 2020, Elsevier Ltd.

In wearable tech, porous TENGs, embedded in garments and footwear, convert biomechanical energy to electrical power, ensuring sustained energy for embedded devices. Such integrations herald advanced potential for next-gen wearables, emphasizing functionality, informativeness, and intelligence. However, the production of textile-based TENGs presents complexities. Doganay et al. fabricated stable, stretchable fiber/fabric-based TENGs using co-axial wet spinning. The fiber’s conductive core comprises a CB, Ag NWs, and TPU composite, with an exposed TPU dielectric sheath (Figure 14a). A 1 cm core–sheath fiber TENG yielded 2 V and 42 nA in V_oc_ and I_sc_, respectively. Integrating this fiber into commercial textiles, they crafted an IoT wristband capable of directing basic computer tasks through Wi-Fi [147]. In another example, Qiu et al. pioneered a self-powered triboelectric nanoskin patch (FNTOP) leveraging eight-layered electrospinning nano/microfiber membranes. Utilizing an innovative all-polymer-based PEDOT:PSS/PVA nanofiber as the electrode and a 3D nano/microtextured TPU as the tribolayer, FNTOP exhibits superior breathability, hydrophobicity, and flexibility (Figure 14b). This enables advanced real-time PSM applications, from force detection to gesture recognition. Consequently, FNTOP signifies a breakthrough for interactive skin devices, health diagnostics, and prosthetic applications, heralding a new era for breathable, skin-integrated electronics [148].

Recognizing the importance of respiration as a key physiological and health marker, especially for sleep disorders, as shown in Figure 14c, Peng et al. introduced a sophisticated real-time respiratory and obstructive sleep apnea hypopnea syndrome (OSAHS) diagnostic system. Employing multilayered polyacrylonitrile and “nylon-66” nanofibers with gold-deposited electrodes, the system achieves a peak power density of 330 mW m^−2^ and an impressive pressure sensitivity of 0.217 kPa^−1^. This innovation showcases immense promise for advanced wearable medical electronics and health surveillance [149]. Wang et al. devised a TENG leveraging shear-thickening fluids (STFs) and magneto-sensitive films, expanding its applications into smart gloves and protective textile electronics with self-powered field monitoring (Figure 14d). This innovative TENG approach holds substantial promise in areas like next-generation energy, smart robotics, and healthcare security [150].

### 4.3. Self-Powering Sensing

**Figure 15:**
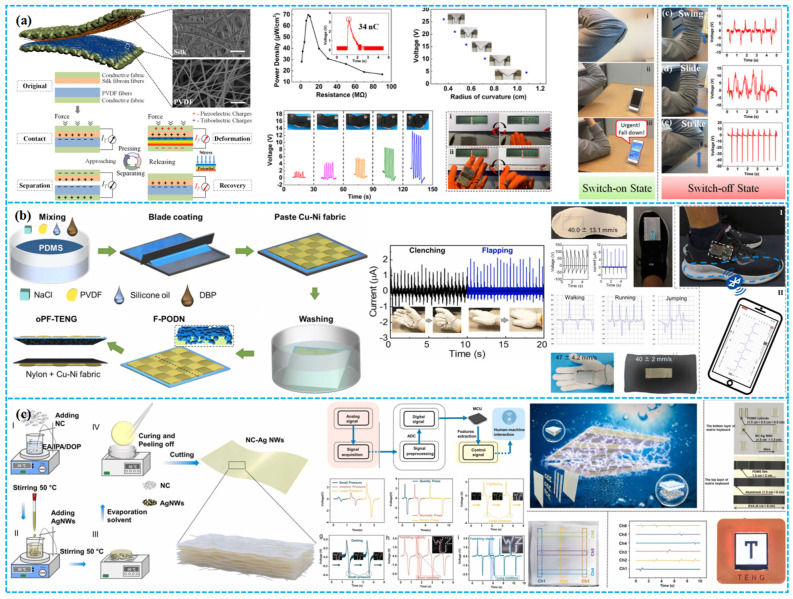
(**a**) Schematic representation of the all-fiber hybrid triboelectric nanogenerator with two electrode layers (conductive fabric) and electrospun silk and PVDF nanofibers as the triboelectric pair. The TPNG, integrated into a breathable fabric, displays omnidirectional force response, evident from its reactions to tapping and bending. Reproduced with permission [151], Copyright 2018, Elsevier Ltd. (**b**) Depiction of the fabrication process of F-PODN and oPF-TENG. Imagery showcases the insole integrated with oPF-TENG, its open-circuit voltage (V_oc_) and short-circuit current (I_sc_), its ability to power 20 LEDs during walking, and its function as a sensor connected to a smartphone via Bluetooth. Reproduced with permission [152], Copyright 2022, Elsevier Ltd. (**c**) Diagrammatic representation of the NC-Ag-NW-based TENG. The structure, signal interpretation, and performance metrics of SNC-TENG are shown, alongside a double electrode 3 × 3 matrix keyboard, displaying real-time sensing signals. Reproduced with permission [153], Copyright 2023, Elsevier Ltd.

In environmental monitoring, porous TENGs offer a sustainable power solution for wireless sensor networks by harnessing ambient vibrations and wind. As shown in Figure 15a, Guo et al. fabricated a hybrid piezoelectric–triboelectric nanogenerator by integrating silk protein and PVDF nanofibers onto conductive textiles. This design combines the flexibility and breathability of textiles, allowing seamless incorporation into garments. Moreover, its capability to recognize body movements via correlated electrical signals highlights significant potential for real-time health monitoring [151].

Tian et al. developed an open-porous PDMS-coated fabric-based TENG (oPF-TENG) using dioctyl phthalate (DBP), NaCl particles, and silicone oil as sacrificial templates. The open-porous PDMS ensures breathability, while the porous structure and PVDF filler optimize its triboelectric efficiency (Figure 15b). The oPF-TENG, with dimensions of 4 × 4 cm^2^, delivers a V_oc_ of ~600 V, I_sc_ of ~15 μA, and a power density of 5.67 W·m^−2^. Demonstrating robustness in energy harvesting and durability in cyclic washing and repeated tests, the oPF-TENG finds potential applications as an energy-harvesting insole and self-powered wearable sensor for motion detection, with inherent breathability for wearer comfort [152]. Zheng et al. developed a nitrocellulose-based TENG with superior waterproofing (WCA: 86.9°), breathability (WVTR: 562.62 g/m^2^/d), and 92% transparency (Figure 15c). Utilizing silver nanowires and the dielectric enhancement effect, the TENG’s triboelectric efficiency increased by 360%. The NC-TENG achieved a power density of 0.38 W/m^2^. Moreover, they introduced a self-powered SNC-TENG sensor for human–machine interaction, adept at capturing both touch pressure and duration, enabling versatile electronic interactions [153].

### 4.4. Human–Machine Interface

**Figure 16:**
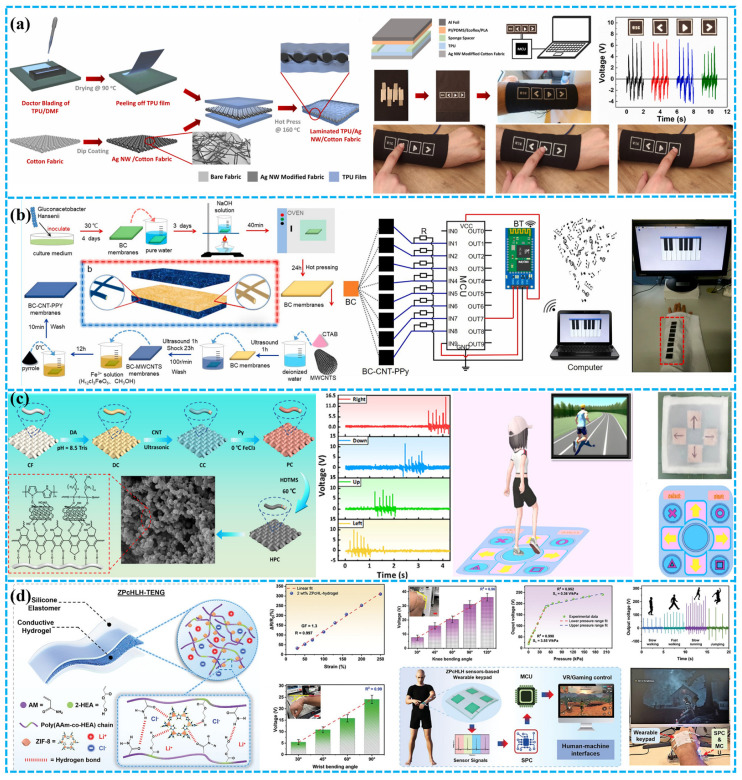
(**a**) Schematic depiction of the fabrication process for TPU-laminated Ag-NW-modified cotton fabrics, including fabrication steps, schematic circuit design, and layered structure. Reproduced with permission [154], Copyright 2021, Elsevier Ltd. (**b**) Schematic representation of the fabrication process for pure BC membrane and conductive BC-CNT-PPy composite membrane. Additionally, a schematic diagram of a wearable electronic piano based on an all-cellulose TENG. Reproduced with permission [155], Copyright 2021, Elsevier Ltd. (**c**) Preparation process and formation mechanism of the HCP composite fabric. The HPC-TENG is affixed to various parts of the human body as a self-powered sensor to monitor motion-induced output voltage. Reproduced with permission [156]. Copyright 2023, American Chemical Society. (**d**) Schematic structure of the ZIF-8@PAAm-co-HEA with LiCl hydrogel-based triboelectric nanogenerator (ZPcHLH-TENG), including the chemical structures and cross-linking networks of ZPcHL hydrogel. Illustration of the self-powered wearable keypad (dimensions: 40 × 40 × 2 mm^3^) serving as a human–machine interface for gaming control. Reproduced with permission [157], Copyright 2023, WILEY-VCH.

Beyond assisting and augmenting human capabilities through sensing and actuation, the pulsed output of wearable TENGs can also serve as an intermediary between humans and machines. While typical human–machine interactions necessitate intricate interfacing, TENGs facilitate interactions autonomously and biocompatibly by harnessing information inherent in the human body. For example, Doganay et al. have introduced a cost-effective and scalable solution to address the aforementioned challenges. They utilized a thermoplastic polyurethane (TPU) film layered with Ag-NW-modified fabric as electrodes in a TENG for self-powering wearable devices in human–machine interaction (Figure 16a). The resulting TENG can be manually tapped to illuminate 185 series-connected green LEDs. Furthermore, the TENG has been adapted into a self-powered electronic wristband with four control buttons, serving as a wearable human–machine interface for controlling fundamental computer operations [154].

Environmental concerns necessitate the utilization of natural materials that are eco-friendly, recyclable, and biodegradable for widespread adoption in TENGs for distributed energy harvesting and wearable self-powered interfaces. As shown in Figure 16b, Zhang et al. have developed an environmentally sustainable and recyclable all-cellulose energy-harvesting and interactive device based on a sandwich-structured BC-TENG. This device comprises pure bacterial cellulose (BC) and conductive BC, serving as the triboelectric layer and electrode, respectively, incorporating conductive and reinforcing nanomaterials. Through the action of cellulase enzymes, BC and BC-CNT-PPy membranes exhibit rapid degradation. The all-cellulose TENG has proven effective in powering commercial electronic devices and has been adapted as a wearable sewn interaction interface to control an electronic piano. This biologically derived TENG holds significant promise for applications in eco-conscious electronics, bioadaptive energy-harvesting systems, wearable human–machine interfaces, and even biomimetic functional artificial electronic organs [155]. He et al. introduced an innovative TENG based on superhydrophobic and conductive fabric (HPC-TENG). By coating cotton fabric with PDA, CNT, PPy, and long-chain carbon silane HDTMS, they have enhanced fabric thickness and triboelectric polarity, resulting in high-output performance, excellent moisture resistance, and electrical conductivity (Figure 16c). The HPC-TENG serves as a pressure sensor for monitoring human movement and a multichannel sensor for intelligent gaming blanket entertainment [156]. As shown in Figure 16d, Rahman et al. have improved the performance of TENGs for wearables by incorporating ZIF-8 as a reinforcing nanofiller in a hydrogel with LiCl electrolyte. This optimized ZIF-8-based hydrogel electrode enhances TENG output, achieving a 3.2-fold increase compared to pure hydrogel-based TENGs. These TENGs efficiently harvest biomechanical energy even at sub-zero temperatures, serving as a reliable power source for small electronic devices and demonstrating exceptional self-powered pressure-sensing capabilities for human–machine interfaces (HMIs) [157].

The TENG traditionally serves as an energy harvester from mechanical motion, finding applications in self-powered sensors, micro/nano power sources, blue energy harvesting, and high-voltage generation. These applications, however, face significant environmental constraints, including humidity, temperature, electromagnetic interference, mechanical flexibility, transparency, breathability, hydrophobicity, and acoustics. Customization of TENG design parameters, such as material properties, structural design, and operational cycles, offers solutions to address these challenges. For instance, controlling pore density and size in porous materials can enhance thermal insulation, rendering TENGs suitable for adverse environmental conditions.

Since the advent of TENGs, foam-based dielectrics have seen significant development. Silicon-based foams with large pores and high elasticity are utilized in pressure sensors. Foams with micro/macroporosity and fibrous dielectrics are preferred for TENG-based pressure sensors with elasticity requirements. Replacing traditional air gaps within TENG structures with foam voids simplifies design. Breathable smart wearables incorporate fibrous dielectrics and porous structural designs from fabrics and yarns, prioritizing breathability, elasticity, stretchability, and flexibility. These features are evident in textile and yarn-based structures and large-pore fibrous TENG materials. Additionally, fibrous electrodes have emerged alongside established fibrous dielectrics for TENG applications, achieved through conductive nanowires or non-conductive fibrous dielectric coatings.

In light of recent significant advancements in hydrogel and aerogel processing, gel-based electrodes and dielectrics for TENGs have garnered substantial attention. These materials exhibit the Knudsen effect within their nanoporous structures, resulting in exceptional thermal insulation properties, rendering them ideal for extreme temperature environments, sound absorption, and environments with high levels of electromagnetic interference. Furthermore, highly porous gels yield optically transparent TENGs, primarily employed in human–machine interface applications, where transparent sensors or electrical devices are seamlessly integrated with the human skin. Key design objectives for TENGs as human–machine interfaces within porous materials include breathability, optical transparency, water repellency, stretchability, and flexibility. In summary, the remarkable attributes of PPMTENGs hold the potential to open up novel applications in self-powered sensors and generators. The output performance of PPMTENGs still needs improvement. In Table 1, we summarize recent research reports on PPMTENGs using polymer porous materials as triboelectric layers and provide an overview of the output performance and mechanical properties of these porous materials and porous structure designs for TENGs.

## 5. Summary and Future Prospects

Porous polymer materials, versatile in composition and adaptable in structure, emerge as pivotal choices for high-performance triboelectrically electroactive layers and conductors. Their advantages encompass flexibility, stretchability, compressibility, permeability, wettability, comfort, biocompatibility, and antimicrobial properties. Porous polymer electrodes and dielectrics have gained substantial attention recently, positioning them as the next-generation materials for TENGs, and promote TENGs as both distributed portable power sources and self-powered sensors. The comprehensive performance of PPMTENGs in various contexts hinges on the fundamental aspects of material design, selection, and manufacturing processes. We provide a systematic review of the latest advancements in porous polymer materials for high-performance TENGs, characterized by multifunctional nanoporous structures exhibiting diversity in geometry, structure, morphology, and performance. Our analysis runs into mechanisms and manufacturing strategies for augmenting triboelectric performance and enhancing triboelectric–electroactive layers. Two primary approaches are distinguished in PPMTENG development: (i) material processing techniques yield porous dielectrics and electrodes, including foam, fiber media, hydrogels, and aerogels, and (ii) structural design innovations introduce air gaps within the PPMTENG, notably within textile and yarn-based configurations. Furthermore, we outline typical PPMTENG applications, emphasizing their roles as energy harvesters, smart wearables, self-powered sensors, and interactive interfaces.

Through materials engineering and structural design, PPMTENGs have exhibited substantial promise in energy harvesting, signal sensing, and medical rehabilitation in recent years. Nevertheless, being an emerging discipline, they also confront enduring hurdles in the essential tradeoff among mechanical properties, electrical properties, and other functionalities. In this discourse, we delve into the orientation of optimization and the future outlook of PPMTENGs, with a specific emphasis on composition and performance considerations.

Leveraging the merits of PPMTENGs, characterized by diverse structures, versatile material options, cost effectiveness, and straightforward fabrication, in conjunction with the advantages inherent to porous foam materials, such as their lightweight nature, extensive specific surface area, and pronounced porosity, presents a viable avenue for the efficient production of PPMTENGs with porous configurations tailored to diverse applications. While the preceding dialogue underscored the prospective uses of PPMTENGs in energy harvesting, self-powered sensors, wearable technology, and intelligent interactive interfaces, it is imperative to acknowledge that there exist notable challenges necessitating innovative solutions to propel the advanced development of PPMTENGs in the future.

The porosity of porous materials is influenced by molecular interactions during the synthesis process, while material assembly plays a critical role in structural design. Various forms of pores with different shapes and sizes can be achieved by altering material or processing factors. Several types of PPMTENG have been reported to meet specific requirements and adjust their performance characteristics accordingly. While the impact of porosity on the triboelectric performance of PPMTENGs has been studied, research on their influence on other properties, such as pore size distribution, remains relatively limited. Furthermore, there is still room for improvement in enhancing triboelectric performance, necessitating further research in the future.

Foam materials with micrometer-sized pores were initially employed as dielectrics. Over time, their high elasticity led to their application in self-powered pressure sensors. The primary requirements for polymer porous structures initially focused on improving triboelectric output but gradually shifted to interface performance criteria, including wettability, biocompatibility, antimicrobial properties, degradability, and stretchability. Functional and conductive films, enhanced by nano/microadditives, have been integrated into all PPMTENGs. TENG technology has advanced intelligent, self-sustaining PPMs for advanced electrical functions and comfortable wearable devices. Subsequently, with the emergence of the IoTs and heightened demands for flexibility, breathability, and stretchability, fiber media and textiles with a broader range and smaller pore sizes have gained widespread use in PPMTENG applications.

Concurrently, to achieve nanoscale porosity, researchers widely employ hydrogels. Porous hydrogels, owing to their exceptional conductivity and ion transport, exhibit significant potential in porous electrode fabrication. Moreover, their compatibility with highly elastic polymers, like silicon-based polymers, enables the realization of transparent, flexible, highly stretchable, and self-healing TENG technology. Recently, nanoscale-structured aerogels offer novel avenues for tailoring various facets of TENG performance, notably antifreezing capabilities. While these methods enhance the TENG’s multifunctionality, emphasizing specific characteristics, such as mechanical flexibility, they may sometimes compromise other aspects, such as triboelectric properties. Hence, innovative approaches, like the creation of graded porous structures, are imperative to simultaneously optimize the TENG’s tribological, mechanical, electrical, thermal, and acoustic performance, making it an ideal choice for practical applications.

The outstanding performance and adjustable properties of porous materials make porous electrodes and dielectrics ideal choices in a wide range of applications, considering various attributes. These attributes encompass sound absorption, thermal insulation, electromagnetic interference (EMI) shielding, water/oil absorption, breathability, optical transparency, conductive ion transport, stretchability, flexibility, hydrophobicity, and elasticity. However, addressing the low dielectric constant of porous dielectrics and the challenges associated with automating and scaling up PPMTENG manufacturing processes will be the primary focus of future research.

In summary, harnessing the inherent advantages of porous structures in wearable devices and biointerface applications, coupled with the rapid advancements in PPMs and TENGs in terms of materials, structure, and functionality, facilitates the seamless integration of PPMTENGs into everyday clothing, skin, and biological interfaces. This development holds promise for a more significant role in next-generation power systems, smart self-powered systems, and interactive scenarios in energy management and information communication.

## Figures and Tables

**Table 1:** Output performance and mechanical properties of PPMTENGs with different porous polymer materials and porous structural design.

Porous Materials and Structure	Material	The Other Electrode	Average Pore Size (μm)	Electrical Output	Application	Ref.
Foam	PDMS	Al	2	130 V/-	Energy harvesting	[74]
Foam	PDMS	Kapton	80	65 V/1 μA	Self-powered active sensing system	[75]
Foam	PDMS	Au	1500	450 V/0.14 mA cm^−2^	Energy harvesting	[76]
Foam	PDMS	Cu	-	291.14 V/35.49 µA	Energy harvesting	[77]
Foam	PTFE	Al	-	5.1 V/6.7 µA	Energy harvesting	[78]
Foam	PDMS	Cu	250	184 V/9.5 μA	Energy harvesting	[79]
Foam	CNT-PDMS	PFA cloth	200	500 V/40 μA	Energy harvesting	[80]
Foam	WPS	PTFE	~4	~250 V/~52 μA	Energy harvesting and self-powered sensor	[81]
Foam	PDMS	PU-GR@MCNTs	-	92 V/-	Energy harvesting and self-powered sensor	[120]
Foam	Ni/PU	PP	300	21 V/0.31 μA	Energy harvester and self-powered sensor	[121]
Foam	PANI/PU	PTFE	100	520 V/6.3 μA	Energy harvesting and ammonia sensing	[122]
Foam	PDMS-CNT/PTFE	Al	250	320 V/13 μA	Energy harvesting	[123]
Foam	Silicon rubber	-	200	24 V/188 nA	Energy harvesting and self-powered sensor	[124]
Foam	PDMS	Cu	500	50 V/400 nA	Energy harvesting	[126]
Foam	CNTs@Fe_3_O_4_/PU	FEP	10	34.8 V/267.1 nA	Energy harvesting	[144]
Foam	PP/PE	PTFE	500	78 V/750 nA	Energy harvesting	[145]
Foam	PI	SRPA	100	48.19 V/1.243 μA	Energy harvesting	[158]
Aerogel	CNF	PDMS	3	60.6 V/7.7 μA	Energy harvesting	[83]
Aerogel	Cellulose II	PTFE	0.01–0.025	65 V/1.86 μA	Energy harvesting	[84]
Aerogel	PBOA	PEO	0.3	40 V	Energy harvesting	[85]
Aerogel	PI	PA	0.686	115 V/9.5 μA	Energy harvesting	[86]
Aerogel	CGD/CNF	-	-	38 V/3 μA	Energy harvesting	[87]
Aerogel	MXene/CMC	PVDF	-	54.37 V/1.22 μA	Energy harvest and self-powered sensing	[67]
Aerogel	BC/HEC	PVDF	77.9	21 V/0.39 μA	Energy harvest and self-powered sensing	[88]
Aerogel	CNF	PDMS	40	75 V/-	Energy harvest and self-powered sensing	[89]
Aerogel	CaNC	FEP	-	80 V/-	Energy harvest and self-powered sensing	[90]
Aerogel	PIA	Liquids	0.2	12 V/300 nA	Biochemical sensing	[91]
Aerogel	CCA	FEP	300	200.4 V/18.2 μA	Self-powered sensing and human–machine interfaces	[125]
Fibrous media	ANF	PVDF	0.85	130 V/12 μA	Energy harvesting	[93]
Fibrous media	Co-NPC	PVDF	0.95	710 V/-	Energy harvest and self-powered sensing	[94]
Fibrous media	PAN/TiO_2_	Nylon	0.5	60 V/0.05 μA	Self-powered sensing and human–machine interfaces	[95]
Fibrous media	PCL/CNT	Ecoflex	0.6	2.24 kV/256 μA	Energy harvesting	[96]
Fibrous media	SPSM	Al	10	36.8 V/0.91 μA	Energy harvest and self-powered sensing	[97]
Fibrous media	MXene/TPU	Al	0.39	20.1 V/0.92 μA	Energy harvest and self-powered sensing	[98]
Fibrous media	SEBC	Cu	200	266 V/5.9 μA	Intelligent wearable devices	[105]
Fibrous media	PVDF-TrFE	MCNT/PDMS	2	508 V/16.5 μA	Energy harvesting	[106]
Fibrous media	PVDF	-	6	54 mV/0.31 nA	Energy harvesting	[107]
Fibrous media	PLGA	PTFE	2	95 V/1.7 μA	Energy harvest and self-powered sensing	[127]
Fibrous media	CB/Ag NW/TPU	PDMS	1000	2 V/42 nA	Intelligent wearable devices	[147]
Fibrous media	PI/TPU	TPU	2	0.92 V/9 nA	Intelligent wearable devices	[148]
Fibrous media	PA66	PAN	2	5.2 V/2 nA	Intelligent wearable devices	[149]
Fibrous media	Silk	PVDF	3	500 V/12 μA	Self-powered sensing	[151]
Textile-based	Cotton/SCNT	Cu	200	0.2 V/0.29 μA	Energy harvest and self-powered sensing	[132]
Textile-based	PDMS	Nitrile	170	397 V/6.8 μA	Energy harvest and self-powered sensing	[133]
Textile-based	Nylon	PDMS-BTO	-	864 V/28.6 μA	Energy harvest and self-powered sensing	[134]
Textile-based	Nylon	E-PTFE	500	800 V/15 μA	Energy harvest and self-powered sensing	[135]
Textile-based	Nitrile,	Silicone rubber	300	-/13 μA	Self-powered sensing and intelligent wearable devices	[136]
Textile-based	PA	PTFE	-	4500 V/40 μA	Energy harvesting	[140]
Textile-based	PET	Parylene	200	130 V/37 µA	Energy harvesting	[146]
Textile-based	PVDF/PDMS	Nylon	100	600 V/15 μA	Energy harvest and self-powered sensing	[152]
Textile-based	TPU	PLA	250	162 V/42 µA	Self-powered sensing and human–machine interfaces	[154]
Textile-based	HPC	PTFE	500	180 V/35 μA	Energy harvest and human–machine interfaces	[156]
Yarn	SUS	PDMS	400	69.5 V/-	Energy harvesting	[141]
Yarn	PE	PDMS	1000	200 V/10 μA	Self-powered sensing and intelligent wearable devices	[142]
Yarn	PI	PTFE	1000	8 V/0.08 μA	Self-powered sensing and intelligent wearable devices	[143]
Yarn	FOTS	Ag	10000	43 V/9.9 μA	Energy harvesting	[159]
Yarn	PDMS	Nylon	500	1.5 V/0.0055 μA	Energy harvest and self-powered sensing	[160]
Yarn	Polyester/cotton	Polyurethane	200	75 V/1.2 μA	Energy harvesting	[161]
Yarn	Dragon Skin	rGP/PDMS	-	10 V/0.6 μA	Self-powered sensing	[162]

## Data Availability

Data available on request from the authors.

## References

[1] Han Y., Wang W., Zou J., Li Z., Cao X., Xu S. (2020). Self-powered energy conversion and energy storage system based on triboelectric nanogenerator. Nano Energy.

[2] Shrestha K., Sharma S., Pradhan G.B., Bhatta T., Maharjan P., Rana S.M.S., Lee S., Seonu S., Shin Y., Park J.Y. (2022). A Siloxene/Ecoflex nanocomposite-based triboelectric nanogenerator with enhanced charge retention by MoS_2_/LIG for self-powered touchless sensor applications. Adv. Funct. Mater..

[3] Lei H., Xiao J., Chen Y., Jiang J., Xu R., Wen Z., Dong B., Sun X. (2022). Bamboo-inspired self-powered triboelectric sensor for touch sensing and sitting posture monitoring. Nano Energy.

[4] Li W., Pei Y., Zhang C., Kottapalli A.G.P. (2021). Bioinspired designs and biomimetic applications of triboelectric nanogenerators. Nano Energy.

[5] Liu Y., Ping J., Ying Y. (2021). Recent progress in 2D-nanomaterial-based triboelectric nanogenerators. Adv. Funct. Mater..

[6] Chang A., Uy C., Xiao X., Chen J. (2022). Self-powered environmental monitoring via a triboelectric nanogenerator. Nano Energy.

[7] Mi Y., Lu Y., Wang X., Zhao Z., Cao X., Wang N. (2022). From Triboelectric nanogenerator to uninterrupted power supply system: The key role of electrochemical batteries and supercapacitors. Batteries.

[8] Hanani Z., Izanzar I., Amjoud M., Mezzane D., Lahcini M., Uršič H., Prah U., Saadoune I., Luk’Yanchuk I.A., Kutnjak Z. (2021). Lead-free nanocomposite piezoelectric nanogenerator film for biomechanical energy harvesting. Nano Energy.

[9] Yu D., Zheng Z., Liu J., Xiao H., Huangfu G., Guo Y. (2021). Super flexible and lead-free piezoelectric nanogenerator as a highly sensitive self-powered sensor for human motion monitoring. Nano-Micro Lett..

[10] Kim W.-G., Kim D.-W., Tcho I.-W., Kim J.-K., Kim M.-S., Choi Y.-K. (2021). Triboelectric Nanogenerator: Structure, Mechanism, and Applications. ACS Nano.

[11] Parandeh S., Etemadi N., Kharaziha M., Chen G., Nashalian A., Xiao X., Chen J. (2021). Advances in Triboelectric Nanogenerators for Self-Powered Regenerative Medicine. Adv. Funct. Mater..

[12] Dong K., Peng X., Cheng R., Wang Z.L. (2022). Smart textile triboelectric nanogenerators: Prospective strategies for improving electricity output performance. Nanoenergy Adv..

[13] Zhou Y., Deng W., Xu J., Chen J. (2020). Engineering materials at the nanoscale for triboelectric nanogenerators. Cell Rep. Phys. Sci..

[14] Chen J., Wang Z.L. (2017). Reviving vibration energy harvesting and self-powered sensing by a triboelectric nanogenerator. Joule.

[15] Liang S., Wang Y., Liu Q., Yuan T., Yao C. (2021). The recent progress in cellulose paper-based triboelectric nanogenerators. Adv. Sustain. Syst..

[16] Dong K., Wang Z.L. (2021). Self-charging power textiles integrating energy harvesting triboelectric nanogenerators with energy storage batteries/supercapacitors. J. Semicond..

[17] Yang X., Liu G., Guo Q., Wen H., Huang R., Meng X., Duan J., Tang Q. (2022). Triboelectric sensor array for internet of things based smart traffic monitoring and management system. Nano Energy.

[18] Zhang C., Lin X., Zhang N., Lu Y., Wu Z., Liu G., Nie S. (2019). Chemically functionalized cellulose nanofibrils-based gear-like triboelectric nanogenerator for energy harvesting and sensing. Nano Energy.

[19] Jin L., Zhang S.L., Xu S., Guo H., Yang W., Wang Z.L. (2021). Free-fixed rotational triboelectric nanogenerator for self-powered real-time wheel monitoring. Adv. Mater. Technol..

[20] Lama J., Yau A., Chen G.R., Sivakumar A., Zhao X., Chen J. (2021). Textile triboelectric nanogenerators for self-powered biomonitoring. J. Mater. Chem. A.

[21] Xuan Z., Wang Z.L., Wang N., Cao X. (2023). Thermal-driven soft-contact triboelectric nanogenerator for energy harvesting and industrial cooling water monitoring. Small.

[22] Wu H., Wang S., Wang Z., Zi Y. (2021). Achieving ultrahigh instantaneous power density of 10 MW/m2 by leveraging the oppo-site-charge-enhanced transistor-like triboelectric nanogenerator (OCT-TENG). Nat. Commun..

[23] Shi X., Chen S., Zhang H., Jiang J., Ma Z., Gong S. (2019). Portable self-charging power system via integration of a flexible paper-based triboelectric nanogenerator and supercapacitor. ACS Sustain. Chem. Eng..

[24] Mi Y.J., Lu Y., Shi Y.L., Zhao Z.Q., Wang X.Q., Meng J.J., Cao X., Wang N. (2023). Biodegradable polymers in triboelectric nano-generators. Polymers.

[25] Tang Y., Zheng Q., Chen B., Ma Z., Gong S. (2017). A new class of flexible nanogenerators consisting of porous aerogel films driven by mechanoradicals. Nano Energy.

[26] Yang J.C., Mun J., Kwon S.Y., Park S., Bao Z., Park S. (2019). Electronic skin: Recent progress and future prospects for skin-attachable devices for health monitoring-robotics, and prosthetics. Adv. Mater..

[27] Chen A., Zhang C., Zhu G., Wang Z.L. (2020). Polymer materials for high-performance triboelectric nanogenerators. Adv. Sci..

[28] Jayababu N., Kim D. (2021). ZnO nanorods@conductive carbon black nanocomposite based flexible integrated system for energy conversion and storage through triboelectric nanogenerator and supercapacitor. Nano Energy.

[29] Rastegardoost M.M., Tafreshi O.A., Saadatnia Z., Ghaffari-Mosanenzadeh S., Park C.B., Naguib H.E. (2023). Recent advances on porous materials and structures for high-performance triboelectric nanogenerators. Nano Energy.

[30] Chen H., Huang J., Liu J., Gu J., Zhu J., Huang B., Bai J., Guo J., Yang X., Guan L. (2021). High toughness multifunctional organic hydrogels for flexible strain and temperature sensor. J. Mater. Chem. A.

[31] Guo X., Yang F., Sun X., Bai Y., Liu G., Liu W., Wang R., He X. (2022). Anti-freezing self-adhesive self-healing degradable touch panel with ultra-stretchable performance based on transparent triboelectric nanogenerators. Adv. Funct. Mater..

[32] Xu Z., Zhou F., Yan H., Gao G., Li H., Li R., Chen T. (2021). Anti-freezing organohydrogel triboelectric nanogenerator toward highly efficient and flexible human-machine interaction at −30 °C. Nano Energy.

[33] Feng J., Su B.L., Xia H., Zhao S., Gao C., Wang L., Ogbeide O., Feng J., Hasan T. (2021). Printed aerogels: Chemistry, processing, and applications. Chem. Soc. Rev..

[34] Chen Y., Zhang L., Yang Y., Pang B., Xu W., Duan G., Jiang S., Zhang K. (2021). Recent progress on nanocellulose aero-gels-preparation, modification, composite fabrication, applications. Adv. Mater..

[35] Wang X., Liang L., Lv H., Zhang Y., Chen G. (2021). Elastic aerogel thermoelectric generator with vertical temperature-difference architecture and compression-induced power enhancement. Nano Energy.

[36] Wang L., Fu X., He J., Shi X., Chen T., Chen P., Wang B., Peng H. (2020). Application challenges in fiber and textile electronics. Adv. Mater..

[37] Jang Y., Kim S.M., Spinks G.M., Kim S.J. (2020). Carbon nanotube yarn for fiber-shaped electrical sensors, actuators, and energy storage for smart systems. Adv. Mater..

[38] Tong Y., Feng Z., Kim J., Robertson J.L., Jia X., Johnson B.N. (2020). 3D printed stretchable triboelectric nanogenerator fibers and devices. Nano Energy.

[39] Li Y., Xiao S., Luo Y., Tian S., Tang J., Zhang X., Xiong J. (2022). Advances in electrospun nanofibers for triboelectric nanogenerators. Nano Energy.

[40] Liu S., Yuan F., Sang M., Zhou J., Zhang J., Wang S., Li J., Xuan S., Gong X. (2021). Functional sponge-based triboelectric nan-ogenerators with energy harvesting, oil–water separating and multi-mode sensing performance. J. Mater. Chem. A.

[41] Zhang C., Chen H., Ding X., Lorestani F., Huang C., Zhang B., Zheng B., Wang J., Cheng H., Xu Y. (2022). Human motion-driven self-powered stretchable sensing platform based on laser-induced graphene foams. Appl. Phys. Rev..

[42] Zhu D., Handschuh-Wang S., Zhou X. (2017). Recent progress in fabrication and application of polydimethylsiloxane sponges. J. Mater. Chem. A.

[43] Tan X.Q., Wang S.T., You Z.Y., Zheng J.M., Liu Y. (2023). High Performance Porous Triboelectric Nanogenerator Based on Silk Fibroin@MXene Composite Aerogel and PDMS Sponge. ACS Materials Lett..

[44] Sun S., Liu Z.-J., Zheng J.-Q., Cheng Q.-K., Tan Y.-L., Huang S.-L., Zhang L., Wang Y.-M., Zhou H.-M. (2023). A Directional Chitosan Sound Sensor Based on Piezoelectric–Triboelectric Sensing. Nano Energy.

[45] Huang J.Y., Hao Y., Zhao M., Li W., Huang F.L., Wei Q.F. (2021). All-fiber-structured triboelectric nanogenerator via one-pot electrospinning for self-powered wearable sensors. ACS Appl. Mater. Interfaces..

[46] Li Y., Yao M.Z., Luo Y.-D., Li J., Wang Z.-L., Liang C., Qin C.-R., Huang C.-X., Yao S.-Q. (2023). Polydopamine-Reinforced Hemicellulose-Based Multifunctional Flexible Hydrogels for Human Movement Sensing and Self-Powered Transdermal Drug Delivery. ACS Appl. Mater. Interfaces.

[47] Feng P.Y., Xia Z.K., Sun B.B., Jing X., Li X., Tao X.M., Mi H.Y., Liu Y.J. (2021). Enhancing the Performance of Fabric-Based Triboelectric Nanogenerators by Structural and Chemical Modification. ACS Appl. Mater. Interfaces.

[48] Ma L., Zhou M., Wu R., Patil A., Gong H., Zhu S., Wang T., Zhang Y., Shen S., Dong K. (2020). Continuous and Scalable Manufacture of Hybridized Nano-Micro Triboelectric Yarns for Energy Harvesting and Signal Sensing. ACS Nano.

[49] Luo J., Wang Z.L. (2019). Recent advances in triboelectric nanogenerator based self-charging power systems. Energy Storage Mater..

[50] Zhang Z., Zhang Q., Zhou Z., Wang J., Kuang H., Shen Q., Yang H. (2022). High-power triboelectric nanogenerators by using in-situ carbon dispersion method for energy harvesting and self-powered wireless control. Nano Energy.

[51] Chen M., Zhou Y., Lang J., Li L., Zhang Y. (2022). Triboelectric nanogenerator and artificial intelligence to promote precision medicine for cancer. Nano Energy.

[52] Li Y., Zheng W., Zhang H., Wang H., Cai H., Zhang Y., Yang Z. (2020). Electron transfer mechanism of graphene/Cu hetero-structure for improving the stability of triboelectric nanogenerators. Nano Energy.

[53] Cheng J., Ding W., Zi Y., Lu Y., Ji L., Liu F., Wu C., Wang Z.L. (2018). Triboelectric microplasma powered by mechanical stimuli. Nat. Commun..

[54] Korkmaz S., Kariper İ.A. (2020). Aerogel based nanogenerators: Production methods, characterizations and applications. Int. J. Energy Res..

[55] Shao Y., Luo C., Deng B.W., Yin B., Yang M.B. (2020). Flexible porous silicone rubber-nanofiber nanocomposites generated by supercritical carbon dioxide foaming for harvesting mechanical energy. Nano Energy.

[56] Xiong J., Cui P., Chen X., Wang J., Parida K., Lin M.F., Lee P.S. (2018). Skin-touch-actuated textile-based triboelectric nanogenerator with black phosphorus for durable biomechanical energy harvesting. Nat. Commun..

[57] Gupta S., Dey M., Matzke C., Ellis G., Javaid S., Hall K., Ji Y., Payne S. (2019). Synthesis and characterization of novel foams by pyrolysis of lignin. TAPPI J..

[58] Jiang W., Li H., Liu Z., Li Z., Tian J., Shi B., Zou Y., Ouyang H., Zhao C., Zhao L. (2018). Fully bioabsorbable natural materials based triboelectric nanogenerators. Adv. Mater..

[59] Biutty M.N., Koo J.M., Zakia M., Handayani P.L., Choi U.H., Yoo S.I. (2020). Dielectric control of porous polydimethylsiloxane elastomers with Au nanoparticles for enhancing the output performance of triboelectric nanogenerators. RSC Adv..

[60] Chun J., Kim J.W., Jung W.S., Kang C.Y., Kim S.W., Wang Z.L., Baik J.M. (2015). Mesoporous pores impregnated with Au nano-particles as effective dielectrics for enhancing triboelectric nanogenerator performance in harsh environments. Energy Environ. Sci..

[61] Sun J., Choi H., Cha S., Ahn D., Choi M., Park S., Cho Y., Lee J., Park T., Park J.J. (2021). Highly enhanced triboelectric per-formance from increased dielectric constant induced by ionic and interfacial polarization for chitosan based multi-modal sensing system. Adv. Funct. Mater..

[62] Mannsfeld S.C.B., Tee B.C.-K., Stoltenberg R.M., Chen C.V.H.-H., Barman S., Muir B.V.O., Sokolov A.N., Reese C., Bao Z. (2010). Highly sensitive flexible pressure sensors with microstructured rubber dielectric layers. Nat. Mater..

[63] Sriphan S., Pharino U., Charoonsuk T., Pulphol P., Pakawanit P., Khamman O., Vittayakorn W., Vittayakorn N., Maluangnont T. (2022). Tailoring charge affinity, dielectric property, and band gap of bacterial cellulose paper by multifunctional Ti_2_NbO_7_ nanosheets for improving triboelectric nanogenerator performance. Nano Res..

[64] Paria S., Si S.K., Karan S.K., Das A.K., Maitra A., Bera R., Halder L., Bera A., De A., Khatua B.B. (2019). A strategy to develop highly efficient TENGs through the dielectric constant, internal resistance optimization, and surface modification. J. Mater. Chem. A.

[65] Haider Z., Haleem A., Ahmad R.U.S., Farooq U., Shi L., Claver U.P., Memon K., Fareed A., Khan I., Mbogba M.K. (2020). Highly porous polymer cryogel based tribopositive material for high performance triboelectric nanogenerators. Nano Energy.

[66] Shanbedi M., Ardebili H., Karim A. (2023). Polymer-based triboelectric nanogenerators: Materials, characterization, and applications. Prog. Polym. Sci..

[67] Cheng Y., Zhu W., Lu X., Wang C. (2022). Lightweight and flexible MXene/carboxymethyl cellulose aerogel for electromagnetic shielding, energy harvest and self-powered sensing. Nano Energy.

[68] Liu Y., Zhang Z., Yang X., Li F., Liang Z., Yong Y., Dai S., Li Z. (2023). A stretchable, environmentally stable, and mechanically robust nanocomposite polyurethane organohydrogel with anti-freezing, anti-dehydration, and electromagnetic shielding properties for strain sensors and magnetic actuators. J. Mater. Chem. A.

[69] Wang Z.L. (2020). Triboelectric nanogenerator (TENG)—Sparking an energy and sensor revolution. Adv. Energy Mater..

[70] Zhu J., Zhu M., Shi Q., Wen F., Liu L., Dong B., Haroun A., Yang Y., Vachon P., Guo X. (2020). Progress in TENG technology—A journey from energy harvesting to nanoenergy and nanosystem. EcoMat.

[71] Wu M., Gao Z., Yao K., Hou S., Liu Y., Li D., He J., Huang X., Song E., Yu J. (2021). Thin, soft, skin-integrated foam-based triboelectric nanogenerators for tactile sensing and energy harvesting. Mater. Today Energy.

[72] Mao Y., Zhao P., McConohy G., Yang H., Tong Y., Wang X. (2014). Sponge-like piezoelectric polymer films for scalable and integratable nanogenerators and self-powered electronic systems. Adv. Energy Mater..

[73] Xia X., Chen J., Guo H., Liu G., Wei D., Xi Y., Wang X., Hu C. (2016). Embedding variable micro-capacitors in polydimethylsiloxane for enhancing output power of triboelectric nanogenerator. Nano Res..

[74] Lee K.Y., Chun J., Lee J., Kim K.N., Kang N., Kim J., Kim M.H., Shin K., Gupta M.K., Baik J.M. (2014). Hydrophobic sponge structure-based triboelectric nanogenerator. Adv. Mater..

[75] Kou H., Wang H., Cheng R., Liao Y., Shi X., Luo J., Li D., Wang Z.L. (2022). Smart pillow based on flexible and breathable tribo-electric nanogenerator arrays for head movement monitoring during sleep. ACS Appl. Mater. Interfaces.

[76] Kim D., Park S.J., Jeon S.B., Seol M.L., Choi Y.K. (2016). A triboelectric sponge fabricated from a cube sugar template by 3D soft lithography for super hydrophobicity and elasticity. Adv. Electron. Mater..

[77] Lu Y., Qin Q., Meng J., Mi Y., Wang X., Cao X., Wang N. (2023). Constructing highly flexible dielectric sponge for enhancing triboelectric performance. Chem. Eng. J..

[78] Wang M., Zhang N., Tang Y., Zhang H., Ning C., Tian L., Li W., Zhang J., Mao Y., Liang E. (2017). Single-electrode triboelectric nanogenerators based on sponge-like porous PTFE thin films for mechanical energy harvesting and self-powered electronics. J. Mater. Chem. A.

[79] Peng Z., Song J., Gao Y., Liu J., Lee C., Chen G., Wang Z., Chen J., Leung M.K. (2021). A fluorinated polymer sponge with superhydrophobicity for high-performance biomechanical energy harvesting. Nano Energy.

[80] Kim W.-G., Kim J.-K., Kim D.-W., Tcho I.-W., Choi Y.-K. (2022). A triboelectric nanogenerator implemented with an acoustic foam for a self-driven silent tire. Nano Energy.

[81] Nawaz S.M., Saha M., Sepay N., Mallik A. (2022). Energy-from-waste: A triboelectric nanogenerator fabricated from waste poly-styrene for energy harvesting and self-powered sensor. Nano Energy.

[82] Zhao G., Shi L., Yang G., Zhuang X., Cheng B. (2022). 3D fibrous aerogels from 1D polymer nanofibers for energy and environmental applications. J. Mater. Chem. A.

[83] Zheng Q., Fang L., Guo H., Yang K., Cai Z., Meador M.A.B., Gong S. (2018). Highly porous polymer aerogel film-based triboelectric nanogenerators. Adv. Funct. Mater..

[84] Zhang L., Liao Y., Wang Y., Zhang S., Yang W., Pan X., Wang Z.L. (2020). Cellulose II Aerogel-Based Triboelectric Nanogenerator. Adv. Funct. Mater..

[85] Qian Z., Li R., Guo J., Wang Z., Li X., Li C., Zhao N., Xu J. (2019). Triboelectric nanogenerators made of poly benzazole aerogels as fire-resistant negative tribo-materials. Nano Energy.

[86] Mi H.-Y., Jing X., Meador M.A.B., Guo H., Turng L.-S., Gong S. (2018). Triboelectric nanogenerators made of porous polyamide nanofiber mats and polyimide aerogel film: Output optimization and performance in circuits. ACS Appl. Mater. Interfaces.

[87] Long S., Feng Y., He F., Zhao J., Bai T., Lin H., Cai W., Mao C., Chen Y., Gan L. (2021). Biomass-derived, multifunctional and wave-layered carbon aerogels toward wearable pressure sensors, supercapacitors and triboelectric nanogenerators. Nano Energy.

[88] Luo C., Ma H., Yu H., Zhang Y., Shao Y., Yin B., Ke K., Zhou L., Zhang K., Yang M.B. (2023). Enhanced triboelectric nanogenerator based on a hybrid cellulose aerogel for energy harvesting and self-powered sensing. ACS Sustain. Chem..

[89] Qian C., Li L., Gao M., Yang H., Cai Z., Chen B., Xiang Z., Zhang Z., Song Y. (2019). All-printed 3D hierarchically structured cellulose aerogel based triboelectric nanogenerator for multi-functional sensors. Nano Energy.

[90] Ahmed A., El-Kady M.F., Hassan I., Negm A., Pourrahimi A.M., Muni M., Selvaganapathy P.R., Kaner R.B. (2019). Fire-retardant, self-extinguishing triboelectric nanogenerators. Nano Energy.

[91] Zhou Q., Wang W., He Y., Li Z., Zhao R., Tao G., Hu B., Hou C. (2023). High-performance polyimide aerogel film-based triboelectric nanogenerator for trace liquid analyzing. ACS Appl. Polym. Mater..

[92] Gao Y., Tian E., Zhang Y., Mo J. (2022). Utilizing electrostatic effect in fibrous filters for efficient airborne particles removal: Principles, fabrication, and material properties. Appl. Mater. Today.

[93] Rastegardoost M.M., Tafreshi O.A., Saadatnia Z., Ghaffari-Mosanenzadeh S., Park C.B., Naguib H.E. (2023). Porous PVDF mats with significantly enhanced dielectric properties and novel dipole arrangement for high-performance triboelectric nanogenerators. Appl. Mater. Today.

[94] Rahman M.T., Rana S.S., Abu Zahed M., Lee S., Yoon E.-S., Park J.Y. (2022). Metal-organic framework-derived nanoporous carbon incorporated nanofibers for high-performance triboelectric nanogenerators and self-powered sensors. Nano Energy.

[95] Jiang Y., Dong K., An J., Liang F., Yi J., Peng X., Ning C., Ye C., Wang Z.L. (2021). UV-Protective, Self-cleaning, and antibacterial nanofiber-based triboelectric nanogenerators for self-powered human motion monitoring. ACS Appl. Mater. Interfaces.

[96] Zhong J., Hou X., He J., Xue F., Yang Y., Chen L., Yu J., Mu J., Geng W., Chou X. (2022). Asymmetric permittivity enhanced bilayer polycaprolactone nanofiber with superior inner interfacial polarization and charge retention for high-output and humidi-ty-resistant triboelectric nanogenerators. Nano Energy.

[97] Li Y., Xiao S., Zhang X., Jia P., Tian S., Pan C., Zeng F., Chen D., Chen Y., Tang J. (2022). Silk inspired in-situ interlocked superelastic microfibers for permeable stretchable triboelectric nanogenerator. Nano Energy.

[98] Hao Y., Zhang Y., Mensah A., Liao S., Lv P., Wei Q. (2023). Scalable, ultra-high stretchable and conductive fiber triboelectric nanogenerator for biomechanical sensing. Nano Energy.

[99] Hu C., Wang F., Cui X., Zhu Y. (2023). Recent progress in textile-based triboelectric force sensors for wearable electronics. Adv. Compos. Hybrid. Mater..

[100] Kwak S.S., Yoon H.J., Kim S.W. (2018). Textile-based triboelectric nanogenerators for self-powered wearable electronics. Adv. Funct. Mater..

[101] Yang B., Xiong Y., Ma K., Liu S., Tao X. (2020). Recent advances in wearable textile-based triboelectric generator systems for energy harvesting from human motion. EcoMat.

[102] Cui X., Wu H., Wang R. (2022). Fibrous triboelectric nanogenerators: Fabrication, integration, and application. J. Mater. Chem. A.

[103] Du X., Zhang K. (2022). Recent progress in fibrous high-entropy energy harvesting devices for wearable applications. Nano Energy.

[104] Dong K., Peng X., Wang Z.L. (2020). Fiber/fabric-based piezoelectric and triboelectric nanogenerators for flexible/stretchable and wearable electronics and artificial intelligence. Adv. Mater..

[105] Chen K., Li Y., Yang G., Hu S., Shi Z., Yang G. (2023). Fabric-based TENG woven with bio-fabricated superhydrophobic bacterial cellulose fiber for energy harvesting and motion detection. Adv. Funct. Mater..

[106] Lee C., Cho C., Oh J.H. (2023). Highly flexible triboelectric nanogenerators with electrospun PVDF-TrFE nanofibers on MWCNTs/PDMS/AgNWs composite electrodes. Compos. Part. B Eng..

[107] Guo Y., Zhang H., Zhong Y., Shi S., Wang Z., Wang P., Zhao Y. (2023). Triboelectric nanogenerator-based near-field electrospinning system for optimizing PVDF fibers with high piezoelectric performance. ACS Appl. Mater. Interfaces.

[108] Shi Q., Dong B., He T., Sun Z., Zhu J., Zhang Z., Lee C. (2020). Progress in wearable electronics/photonics—Moving toward the era of artificial intelligence and internet of things. InfoMat.

[109] Parandeh S., Kharaziha M., Karimzadeh F., Hosseinabadi F. (2020). Triboelectric nanogenerators based on graphene oxide coated nanocomposite fibers for biomedical applications. Nanotechnology.

[110] Ge X., Hu N., Yan F., Wang Y. (2023). Development and applications of electrospun nanofiber-based triboelectric nanogenerators. Nano Energy.

[111] Yang W., Cao R., Zhang X., Li H., Li C. (2018). Air-Permeable and Washable Paper–Based Triboelectric Nanogenerator Based on Highly Flexible and Robust Paper Electrodes. Adv. Mater. Technol..

[112] Cao W.T., Ouyang H., Xin W., Chao S., Ma C., Li Z., Chen F., Ma M.G. (2020). A stretchable high output triboelectric nanogenerator improved by MXene liquid electrode with high electronegativity. Adv. Funct. Mater..

[113] Li G., Zhang J., Huang F., Wu S., Wang C.H., Peng S. (2021). Transparent, stretchable and high-performance triboelectric nano-generator based on dehydration-free ionically conductive solid polymer electrode. Nano Energy.

[114] Li Z., Li C., Sun W., Bai Y., Li Z., Deng Y. (2023). A Controlled biodegradable triboelectric nanogenerator based on PEGDA/Laponite hydrogels. ACS Appl. Mater. Interfaces.

[115] Li K., Zhang D., Zhang H., Wang D., Xu Z., Cai H., Xia H. (2023). Triboelectric nanogenerators based on super-stretchable con-ductive hydrogels with the assistance of deep-learning for handwriting recognition. ACS Appl. Mater. Interfaces.

[116] Chen P., Wang Q., Wan X., Yang M., Liu C., Xu C., Hu B., Feng J., Luo Z. (2021). Wireless electrical stimulation of the vagus nerves by ultrasound-responsive programmable hydrogel nanogenerators for anti-inflammatory therapy in sepsis. Nano Energy.

[117] Zhang H., Zhang D., Wang Z., Xi G., Mao R., Ma Y., Wang D., Tang M., Xu Z., Luan H. (2023). Ultrastretchable, self-healing conductive hydrogel-based triboelectric nanogenerators for human-computer interaction. ACS Appl. Mater. Interfaces.

[118] Wu Y., Luo Y., Cuthbert T.J., Shokurov A.V., Chu P.K., Feng S., Menon C. (2022). Hydrogels as Soft Ionic Conductors in Flexible and Wearable Triboelectric Nanogenerators. Adv. Sci..

[119] Panwar V., Babu A., Sharma A., Thomas J., Chopra V., Malik P., Rajput S., Mittal M., Guha R., Chattopadhyay N. (2021). Tunable, conductive, self-healing, adhesive and injectable hydrogels for bioelectronics and tissue regeneration applications. J. Mater. Chem. B.

[120] Li X., Jiang C., Zhao F., Lan L., Yao Y., Yu Y., Ping J., Ying Y. (2019). Fully stretchable triboelectric nanogenerator for energy harvesting and self-powered sensing. Nano Energy.

[121] Cui X., Zhao T., Yang S., Xie G., Zhang Z., Zhang Y., Sang S., Lin Z.H., Zhang W., Zhang H. (2020). A spongy electrode-brush-structured dual-mode triboelectric nanogenerator for harvesting mechanical energy and self-powered trajectory tracking. Nano Energy.

[122] Liu Y., Zheng Y., Wu Z., Zhang L., Sun W., Li T., Wang D., Zhou F. (2021). Conductive elastic sponge-based triboelectric nano-generator (TENG) for effective random mechanical energy harvesting and ammonia sensing. Nano Energy.

[123] Kim W.G., Kim D., Jeon S.B., Park S.J., Tcho I.W., Jin I.K., Han J.K., Choi Y.K. (2018). Multidirection and multiamplitude triboelectric nanogenerator composed of porous conductive polymer with prolonged time of current generation. Adv. Energy Mater..

[124] Park J., Kim I., Yun J., Kim D. (2021). Liquid-metal embedded sponge-typed triboelectric nanogenerator for omnidirectionally de-tectable self-powered motion sensor. Nano Energy.

[125] Wang Z., Chen C., Fang L., Cao B., Tu X., Zhang R., Dong K., Lai Y.-C., Wang P. (2023). Biodegradable, conductive, moisture-proof, and dielectric enhanced cellulose-based triboelectric nanogenerator for self-powered human-machine interface sensing. Nano Energy.

[126] Li Z., Hu K., Yang M., Zou Y., Yang J., Yu M., Wang H., Qu X., Tan P., Wang C. (2019). Elastic Cu@PPy sponge for hybrid device with energy conversion and storage. Nano Energy.

[127] Peng X., Dong K., Ye C., Jiang Y., Zhai S., Cheng R., Liu D., Gao X., Wang J., Wang Z.L. (2020). A breathable, biodegradable, antibacterial, and self-powered electronic skin based on all-nanofiber triboelectric nanogenerators. Sci. Adv..

[128] Chen G., Li Y., Bick M., Chen J. (2020). Smart textiles for electricity generation. Chem. Rev..

[129] Seyedin S., Uzun S., Levitt A., Anasori B., Dion G., Gogotsi Y., Razal J.M. (2020). MXene composite and coaxial fibers with high stretchability and conductivity for wearable strain sensing textiles. Adv. Funct. Mater..

[130] Weng W., Chen P., He S., Sun X., Peng H. (2016). Smart electronic textiles. Angew. Chem. Int. Ed. Engl..

[131] Yan L., Mi Y., Lu Y., Qin Q., Wang X., Meng J., Liu F., Wang N., Cao X. (2022). Weaved piezoresistive triboelectric nanogenerator for human motion monitoring and gesture recognition. Nano Energy.

[132] Lv T., Cheng R., Wei C., Su E., Jiang T., Sheng F., Peng X., Dong K., Wang Z.L. (2023). All-fabric direct-current triboelectric nanogenerators based on the Tribovoltaic effect as power textiles. Adv. Energy Mater..

[133] Sreeja Sadanandan K., Saadi Z., Murphy C., Grikalaite I., Craciun M.F., Neves A.I.S. (2023). Fabric-based triboelectric nanogenerators with ultrasonic spray coated graphene electrodes. Nano Energy.

[134] Gao Y., Xu B., Tan D., Li M., Wang Y., Yang Y. (2023). Asymmetric-elastic-structure fabric-based triboelectric nanogenerators for wearable energy harvesting and human motion sensing. Chem. Eng. J..

[135] Huang T., Zhang J., Yu B., Yu H., Long H., Wang H., Zhang Q., Zhu M. (2019). Fabric texture design for boosting the performance of a knitted washable textile triboelectric nanogenerator as wearable power. Nano Energy.

[136] He T., Wang H., Wang J., Tian X., Wen F., Shi Q., Ho J.S., Lee C. (2019). Self-sustainable wearable textile nano-energy nano-system (NENS) for next-generation healthcare applications. Adv. Sci..

[137] Zhou Z., Padgett S., Cai Z., Conta G., Wu Y., He Q., Zhang S., Sun C., Liu J., Fan E. (2020). Single-layered ultra-soft washable smart textiles for all-around ballistocardiograph, respiration, and posture monitoring during sleep. Biosens. Bioelectron..

[138] Gao Y., Li Z., Xu B., Li M., Jiang C., Guan X., Yang Y. (2022). Scalable core–spun coating yarn-based triboelectric nanogenerators with hierarchical structure for wearable energy harvesting and sensing via continuous manufacturing. Nano Energy.

[139] Dong K., Wu Z., Deng J., Wang A.C., Zou H., Chen C., Hu D., Gu B., Sun B., Wang Z.L. (2018). A Stretchable yarn embedded triboelectric nanogenerator as electronic skin for biomechanical energy harvesting and multifunctional pressure sensing. Adv. Mater..

[140] Chen C., Guo H., Chen L., Wang Y.C., Pu X., Yu W., Wang F., Du Z., Wang Z.L. (2020). Direct current fabric triboelectric nan-ogenerator for biomotion energy harvesting. ACS Nano.

[141] Ko W.B., Choi D.S., Lee C.H., Yang J.Y., Yoon G.S., Hong J.P. (2017). Hierarchically nanostructured 1d conductive bundle yarn-based triboelectric nanogenerators. Adv. Mater..

[142] Cheng M., Liu X., Li Z., Zhao Y., Miao X., Yang H., Jiang T., Yu A., Zhai J. (2023). Multiple textile triboelectric nanogenerators based on UV-protective, radiative cooling, and antibacterial composite yarns. Chem. Eng. J..

[143] Xing F., Ou Z., Gao X., Chen B., Wang Z.L. (2022). Harvesting Electrical energy from high temperature environment by aerogel nano-covered triboelectric yarns. Adv. Funct. Mater..

[144] Zheng J., Wei X., Li Y., Dong W., Li X., Wu Z., Wen J. (2021). Stretchable polyurethane composite foam triboelectric nanogenerator with tunable microwave absorption properties at elevated temperature. Nano Energy.

[145] Li C.L., Song W.Z., Sun D.J., Zhang M., Zhang J., Chen Y.Q., Ramakrishna S., Long Y.Z. (2023). A self-priming air filtration system based on triboelectric nanogenerator for active air purification. Chem. Eng. J..

[146] Pu X., Song W., Liu M., Sun C., Du C., Jiang C., Huang X., Zou D., Hu W., Wang Z.L. (2016). Wearable power-textiles by integrating fabric triboelectric nanogenerators and fiber-shaped dye-sensitized solar cells. Adv. Energy Mater..

[147] Doganay D., Demircioglu O., Cugunlular M., Cicek M.O., Cakir O., Kayaci H.U., Aygün S., Unalan H.E. (2023). Wet spun core-shell fibers for wearable triboelectric nanogenerators. Nano Energy.

[148] Qiu Y., Fang H., Guo J., Wu H. (2022). Fully nano/micro-fibrous triboelectric on-skin patch with high breathability and hydrophobicity for physiological status monitoring. Nano Energy.

[149] Peng X., Dong K., Ning C., Cheng R., Yi J., Zhang Y., Sheng F., Wu Z., Wang Z.L. (2021). All-nanofiber self-powered skin-interfaced real-time respiratory monitoring system for obstructive sleep apnea-hypopnea syndrome diagnosing. Adv. Funct. Mater..

[150] Wang S., Liu S., Zhou J., Li F., Li J., Cao X., Li Z., Zhang J., Li B., Wang Y. (2020). Advanced triboelectric nanogenerator with multi-mode energy harvesting and anti-impact properties for smart glove and wearable e-textile. Nano Energy.

[151] Guo Y., Zhang X.-S., Wang Y., Gong W., Zhang Q., Wang H., Brugger J. (2018). All-fiber hybrid piezoelectric-enhanced triboelectric nanogenerator for wearable gesture monitoring. Nano Energy.

[152] Tan D., Xu B., Gao Y., Tang Y., Liu Y., Yang Y., Li Z. (2022). Breathable fabric-based triboelectric nanogenerators with open-porous architected polydimethylsiloxane coating for wearable applications. Nano Energy.

[153] Zheng T., Li G., Zhang L., Sun W., Pan X., Chen T., Wang Y., Zhou Y., Tian J., Yang Y. (2023). A waterproof, breathable nitro-cellulose-based triboelectric nanogenerator for human-machine interaction. Nano Energy.

[154] Doganay D., Cicek M.O., Durukan M.B., Altuntas B., Agbahca E., Coskun S., Unalan H.E. (2021). Fabric based wearable triboelectric nanogenerators for human machine interface. Nano Energy.

[155] Zhang J., Hu S., Shi Z., Wang Y., Lei Y., Han J., Xiong Y., Sun J., Zheng L., Sun Q. (2021). Eco-friendly and recyclable all cellulose triboelectric nanogenerator and self-powered interactive interface. Nano Energy.

[156] He J., Xue Y., Liu H., Li J., Liu Q., Zhao Y., Mu L., Sun C.L., Qu M. (2023). Humidity-resistant, conductive fabric-based triboelectric nanogenerator for efficient energy harvesting and human–machine interaction sensing. ACS Appl. Mater. Interfaces.

[157] Rahman M.T., Rahman S., Kumar H., Kim K., Kim S. (2023). Metal-organic framework reinforced highly stretchable and durable conductive hydrogel-based triboelectric nanogenerator for biomotion sensing and wearable human-machine interfaces. Adv. Funct. Mater..

[158] Cho H., Jo S., Kim I., Kim D. (2021). Film-Sponge-coupled triboelectric nanogenerator with enhanced contact area based on direct ultraviolet laser ablation. ACS Appl. Mater. Interfaces.

[159] Kim K., Song G., Park C., Yun K.-S. (2017). Multifunctional woven structure operating as triboelectric energy harvester, capacitive tactile sensor array, and piezoresistive strain sensor array. Sensors.

[160] Li X., Lin Z.-H., Cheng G., Wen X., Liu Y., Niu S., Wang Z.L. (2014). 3D Fiber-based hybrid nanogenerator for energy harvesting and as a self-powered pressure sensor. ACS Nano.

[161] Yu A., Pu X., Wen R., Liu M., Zhou T., Zhang K., Zhang Y., Zhai J., Hu W., Wang Z.L. (2017). Core–shell-yarn-based triboelectric nanogenerator textiles as power cloths. ACS Nano.

[162] Fu K., Zhou J., Wu H., Su Z. (2021). Fibrous self-powered sensor with high stretchability for physiological information monitoring. Nano Energy.

